# A new nanomagnetic Pd-Co bimetallic alloy as catalyst in the Mizoroki–Heck and Buchwald–Hartwig amination reactions in aqueous media

**DOI:** 10.1038/s41598-021-95931-6

**Published:** 2021-08-23

**Authors:** Sara Sobhani, Hamed Zarei, José Miguel Sansano

**Affiliations:** 1 Department of Chemistry, College of Sciences, University of Birjand, Birjand, Iran; 2 Departamento de Química Orgánica, Facultad de Ciencias, Centro de Innovación en Química Avanzada (ORFEO-CINQA) and Instituto de Síntesis Orgánica (ISO), Universidad de Alicante, Apdo. 99, 03080 Alicante, Spain

**Keywords:** Chemistry, Organic chemistry, Synthetic chemistry methodology

## Abstract

A Pd-Co bimetallic alloy encapsulated in melamine-based dendrimer supported on magnetic nanoparticles denoted as γ-Fe_2_O_3_@MBD/Pd-Co was synthesized by a facile co-complexation-reduction method and characterized sufficiently. The catalytic evaluation of γ-Fe_2_O_3_@MBD/Pd-Co showed promising results in the Mizoroki–Heck and Buchwald–Hartwig amination reactions of various iodo-, bromo- and challenging chloroarenes in aqueous media. The synergetic cooperative effect of both Pd and Co and dispersion of the catalyst in water due to the encapsulation of γ-Fe_2_O_3_ by melamine-based dendrimer lead to high catalytic performance compared with the monometallic counterparts. The dispersion of the magnetic catalyst also facilitates the recovery and reuse of the catalyst by ten consecutive extraction and final magnetic isolation with no loss of catalytic activity, keeping its structure unaltered.

## Introduction

Haloarenes are frequently transformed into a variety of valuable compounds catalyzed by transition metals such as palladium, copper, nickel, and cobalt with various ligands through cross-coupling reactions^1–4^. Concerning the catalytic metals employed, palladium is the most versatile element in industrial and academic research playing the most prominent part in the removal of halogen atom from halogenated organic compounds. Therefore, since the pioneering works, the homogeneous palladium complexes have attracted the interest of scientists to address coupling reactions due to their excellent functional group tolerance, as well as their excellent catalytic efficiency^5^. However, application of homogeneous palladium catalysts has some limitations such as non-reusability, high cost, poisoning and losing activity, which are important from economic and environmental viewpoints. These problems can be largely overcome by supporting Pd nanoparticles (NPs) or complexes on varied solid supports^6^. Moreover, considering the consumption of expensive Pd, the development of bimetallic nanoparticles of non-noble metal elements with Pd can not only reduce costs, but also stabilizes active Pd species, and improves its resistance to poisoning^7^. The supported bimetallic nanoparticles with two special advantages of using of a lower quantity of Pd and the recovery of the heterogeneous NPs by filtration from the reaction media, could be used as suitable catalysts in cross-coupling reactions. Moreover, because of the synergistic effect, the catalytic activity and selectivity obtained over bimetallic nanoparticles were much higher than that obtained over the monometallic counterparts. Along this line, Pd-based bimetallic nanoparticles including Pd–Ag, Pd-Cu, Pd-Co, Pd-Au or Pd–Ni, supported on different solid substances have been reported^7–11^. Although heterogeneous catalysts can be recovered by filtration or centrifugation methods, these methods are time consuming, and result the impurities in the product due to the loss of the catalyst particles.

In recent decades, magnetic nanoparticles (MNPs), which have been widely surveyed towards many medical and biological uses, have emerged as modern and attractive materials for the supporting of catalytically active species. The magnetically immobilized catalysts can be isolated from the reaction mixture employing magnets^12,13^. This separation is operationally very simple, economic and promising for industrial applications. Moreover, MNPs possess exclusive physical properties, for example, high surface area, surface modification ability and extraordinary thermal and chemical stabilities. However, MNPs suffer from aggregation during the catalytic processes, resulting in a sensible decrease in their catalytic efficiency. Therefore, encapsulation of MNPs using noble metals, carbon, silica and biopolymers stabilize and protect them from oxidation and agglomeration^14,15^.

Dendrimers are a relatively novel class of polymers with a well-defined highly branched, three-dimensional structure and are being used for encapsulation of MNPs^16^. In the dendrimers, the existence of several inner and outer functional groups allows the coordination with transition metals contributing to their stabilization. Because of this property, dendrimers are principally suitable host for metal catalysts. Nanometals are encapsulated within the dendrimer cavities, so that their agglomeration is circumvented and the well-dispersed nanoparticles are obtained. Moreover, the existence of a large number of cavities in dendritic polymers, could absorb and concentrate reactants, and makes the reaction proceed more efficiently. Importantly, the substrates can still simply contact with the encapsulated catalytically active nanoparticle in the dendrimers. As the melamine is rich with nitrogen and contains triazine rings with high stability, melamine-based dendrimers (MBD) with abundant metal-binding nitrogen groups have aroused a lot of interest recently for the incorporation of metal nanoparticles for using in catalytic reactions^17–19^.

In our continuous interest in developing a greener catalyzed reaction^4,20–28^, herein, a melamine-based dendrimer was built to the 1.5-generation onto the γ-Fe_2_O_3_ surface employing a divergent method to synthesize γ-Fe_2_O_3_@melamine-based dendrimer (γ-Fe_2_O_3_@MBD). Bimetallic Pd-Co alloy nanoparticles were then attached to γ-Fe_2_O_3_@melamine-based dendrimer via a co-complexation method followed by reduction with sodium borohydide. This novel catalyst was fully analyzed by several instrumental techniques and applied as a water-dispersible/magnetically reusable palladium-cobalt catalyst for the Mizoroki–Heck and Buchwald–Hartwig amination reactions in aqueous media. The synergistic cooperative effect of both Pd and Co in the catalyst lead to high catalytic performance in the cross-coupling reactions in aqueous media.

## Experimental section

### Materials and methods

Chemicals were purchased from Merck Chemical Company. NMR spectra were recorded on a Bruker Avance DPX-400 and 300 using deuterated CDCl_3_ and DMSO-d_6_ as solvent and TMS as internal standard. The purity of the products and the progress of the reactions were accomplished by TLC on silica-gel polygram SILG/UV254 plates. TEM analysis was performed using TEM microscope (Philips EM 208S). EDS mapping were found using a TSCAN MIRA3. FT-IR spectra were recorded on a Shimadzu Fourier Transform Infrared Spectrophotometer (FT-IR-8300). Thermal gravimetric analysis (TGA) was performed using a Shimadzu thermos-gravimetric analyzer (TG-50). X-ray diffraction (XRD) was done on a Bruker D8-advance X-ray diffractometer with Cu K_α_ (λ = 0.154 nm) radiation. XPS analyses were performed using a VG-Microtech Multilab 3000 spectrometer, equipped with an Al anode. The deconvolution of spectra was carried out by using Gaussian–Lorentzian curves. The Pd and Co content on the catalyst was determined by OPTIMA 7300DV ICP analyzer. Qualitative elemental analysis was determined and analyzed by CHNO elemental analyzer (Thermo Finnigan, FLASH EA 1112 series, Italy).

### Synthesis of γ-Fe_2_O_3_-melamine

Chloro-functionalized γ-Fe_2_O_3_^20^ (1.5 g) was sonicated with Et_3_N (10 mL) and melamine (1.5 mmol, 0.18 g) for 30 min. The mixture was refluxed for 48 h at 60 °C. The resulting light-brown solid was isolated by an external magnet and washed several times with distilled H_2_O (3 × 20 mL) and EtOH (3 × 20 mL). It was dried in a vacuum oven at 50 °C. Elemental analysis of γ-Fe_2_O_3_-melamine for nitrogen (9.88%) showed that 1.2 mmol of melamine was loaded on 1 g of γ-Fe_2_O_3_-melamine.

### Synthesis of γ-Fe_2_O_3_-MBD

The synthesized γ-Fe_2_O_3_-melamine from the previous step (1 g) and Et_3_N (10 mL) was sonicated about 30 min at room temperature. ECH (1 mL) was added to this stirring mixture drop wise. The mixture was heated to a temperature of 60 °C and kept for 24 h. Then, melamine (5 mmol, 0.63 g) was added and further stirred for 48 h at the same temperature. The resulting solid was isolated by an external magnet and washed several times with distilled H_2_O (3 × 20 mL) and EtOH (3 × 20 mL). It was dried in a vacuum oven at 50 °C. Elemental analysis of γ-Fe_2_O_3_-MBD for nitrogen (17.5%) showed that in total, 2 mmol of melamine was loaded on 1 g of γ-Fe_2_O_3_-MBD.

### Synthesis of γ-Fe_2_O_3_-MBD/Pd-Co

A mixture of palladium acetate (0.1 g in 3.0 mL H_2_O) and γ-Fe_2_O_3_-MBD (0.2 g in 10.0 mL) sonicated for 20 min. A solution of CoCl_2_⋅6H_2_O (0.741, 0.529, 0.105 g in 3.0 mL H_2_O) was added to the resulting mixture and sonicated for further 20 min. The pH of the sonicated mixture was controlled between 8 and 10 using sodium hydroxide solution (0.3 M). Then, a solution of sodium borohydride (1.0 M, 10.5 mL) was added to the reaction mixture and stirred for 24 h at ambient temperature. The resulting black solid was separated from aqueous media using an external magnet, washed well with distilled H_2_O (3 × 20 mL) and EtOH (3 × 20 mL) and dried under vacuum at 50 °C for 4 h.

### General procedure for the Mizoroki–Heck cross-coupling reaction catalyzed by γ-Fe_2_O_3_-MBD/Pd-Co

γ-Fe_2_O_3_-MBD/Pd-Co (0.05 mol% based on Pd) was added to a stirred suspension of haloarene (1 mmol), Et_3_N (2 mmol), alkene (1.3 mmol) in water (1 mL). The resulting mixture was heated at 60 °C. The reaction was monitored by TLC and, after the times shown in Table 3, the reaction was cooled down to room temperature. The organic compound was extracted twice with EtOAc (2 × 5 mL). The final organic layer was dried over MgSO_4_ and filtered. Organic solvent evaporated under vacuum to give the crude product, which was purified by column chromatography (silica gel) using 50:1 volume ratio of *n*-hexane:EtOAc as eluent. The aqueous phase, containing the bimetallic catalyst, was again used for a new identical process.

### General procedure for the Buchwald–Hartwig amination reaction of aryl iodides with arylamines catalyzed by γ-Fe_2_O_3_-MBD/Pd-Co

γ-Fe_2_O_3_-MBD/Pd-Co (0.05 mol% based on Pd) was added to a stirred mixture of iodoarene (1 mmol), *t*-BuONa (2 mmol), and the arylamine (1.2 mmol) in H_2_O (1 mL). The resulting suspension was heated at 50 °C and the reaction was monitored by TLC. After the times depicted in Table 6, the reaction mixture was cooled down. The organic product was extracted three times with EtOAc (3 × 5 mL). The combined organic layers were dried over MgSO_4_ and the solvent evaporated under vacuum to give the crude product, which was purified by column chromatography (silica gel) using 10:1 volume ratio of *n-*hexane:EtOAc as eluent. The aqueous phase, containing the bimetallic catalyst, was again used for a new identical process.

### General procedure for the Buchwald–Hartwig amination reaction of aryl chlorides and bromides with arylamines catalyzed by γ-Fe_2_O_3_-MBD/Pd-Co

γ-Fe_2_O_3_-MBD/Pd-Co (0.07 mol% based on Pd) was added to a stirred suspension of chloroarenes or bromoarenes (1 mmol), *t*-BuONa (2 mmol), and arylamines (1.2 mmol) in H_2_O (1 mL). The resulting mixture was heated at 70 °C. The reaction was monitored by TLC and, after the times shown in Table 6, the reaction was cooled down to room temperature. The organic product was extracted three times with EtOAc (5 mL). The combined organic layers were dried over MgSO_4_ and the solvent was evaporated under vacuum to give the crude product, which was purified by column chromatography (silica gel) using 10:1 volume ratio of *n-*hexane:EtOAc as eluent. The aqueous phase, containing the bimetallic catalyst, was again used for a new identical process.

## Results and discussion

**Scheme 1:**
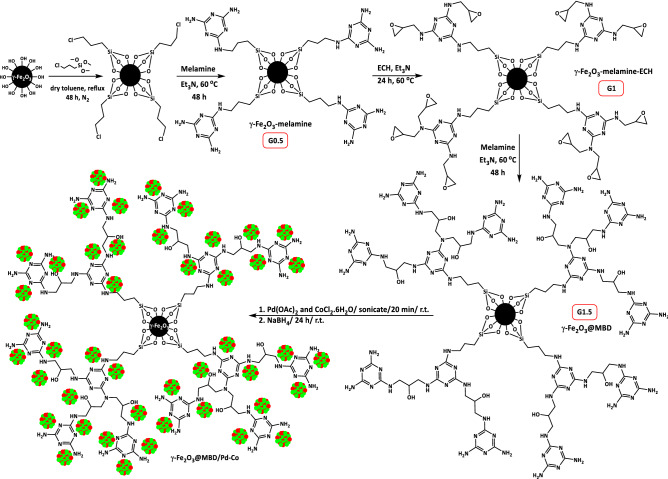
Synthesis of γ-Fe_2_O_3_@MBD/Pd-Co.

In Scheme 1, the approach which we have used for the preparation of Pd/Co bimetallic catalyst (γ-Fe_2_O_3_@MBD/Pd-Co) is illustrated. In the first step, γ-Fe_2_O_3_ was functionalized by the reaction with 3-chloro-trimethoxypropylsilane and subsequent treatment with melamine to produce γ-Fe_2_O_3_-melamine (Scheme 1, **G0.5**). The nucleophilic reaction of γ-Fe_2_O_3_-melamine with epichlorohydrin (ECH), as a bifunctional molecule for growing of dendritic branches afforded γ-Fe_2_O_3_-melamine-ECH (Scheme 1, G1). Ring opening reaction of terminal epoxides in γ-Fe_2_O_3_-melamine-ECH by melamine produced 1.5 generation of dendrimer-magnetite incorporation (γ-Fe_2_O_3_@MBD, **G1.5**). The last step involves the intercalating of metal complexes into the interior cavity of γ-Fe_2_O_3_@MBD followed by reduction with NaBH_4_ as a reducing agent.

**Figure 1:**
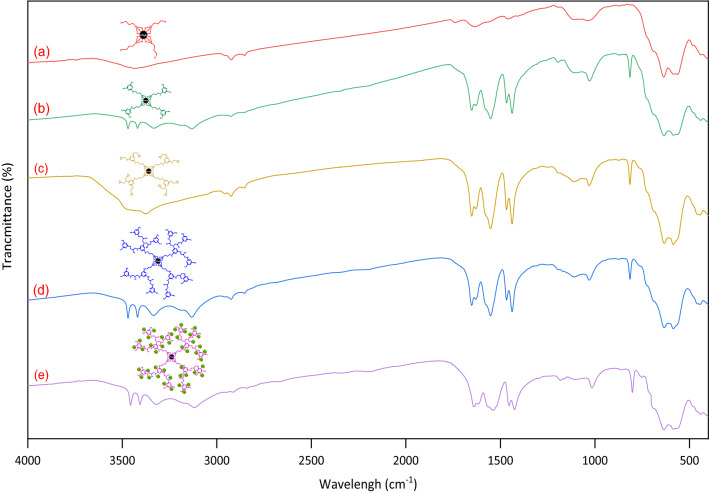
Plots of FT-IR spectra of (**a**) chlorofunctionalized γ-Fe_2_O_3_, (**b**) γ-Fe_2_O_3_-melamine, (**c**) γ-Fe_2_O_3_-melamine-ECH, (**d**) γ-Fe_2_O_3_@MBD and (**e**) γ-Fe_2_O_3_@MBD/Pd-Co.

Chemical structures of all the new synthesized compounds (chlorofunctionalized γ-Fe_2_O_3_, γ-Fe_2_O_3_-melamine, γ-Fe_2_O_3_-melamine-ECH, γ-Fe_2_O_3_@MBD and γ-Fe_2_O_3_@MBD/Pd-Co) were determined via FT-IR spectroscopy (Fig. 1). FT-IR spectra of these compounds exhibited characteristic bands at around 563–636, 1037 and 3437 cm^−1^ attributed to Fe–O, Si–O and O–H bonds, respectively. The FT-IR spectra of γ-Fe_2_O_3_-melamine (Fig. 1b), γ-Fe_2_O_3_@MBD (Fig. 1d) and γ-Fe_2_O_3_@MBD/Pd-Co (Fig. 1e) exhibited typical bands at around 3471, 3415, 1651, 1550 and 814 cm^−1^ attributed to N–H stretching and bending vibrations. In the FT-IR spectrum of γ-Fe_2_O_3_-melamine-ECH (Fig. 1c), N–H band of primary amine vanished and new broad bands at 3471 and 3376 cm^−1^ appeared. The new peaks were represented by the stretching vibration of N–H and O–H groups and proved that melamine and epichlorohydrine were effectively reacted. A slight shifting and variations in the amplitude of the N–H and C=N bands of triazine in Fig. 1e can justify the interaction of the metallic elements with the nitrogen atoms in the catalyst.

The metal content of γ-Fe_2_O_3_@MBD/Pd-Co was calculated and quantified employing ICP analysis revealing 0.75 and 3.15 mmol of Pd and Co, respectively, (molar ratio Pd:Co = 1:4.2) per 1 g of the catalyst. Elemental analysis of γ-Fe_2_O_3_-melamine and γ-Fe_2_O_3_@MBD showed that the loadings of melamine on the catalyst were 9.88 and 17.50%, respectively, based on the nitrogen content.

**Figure 2:**
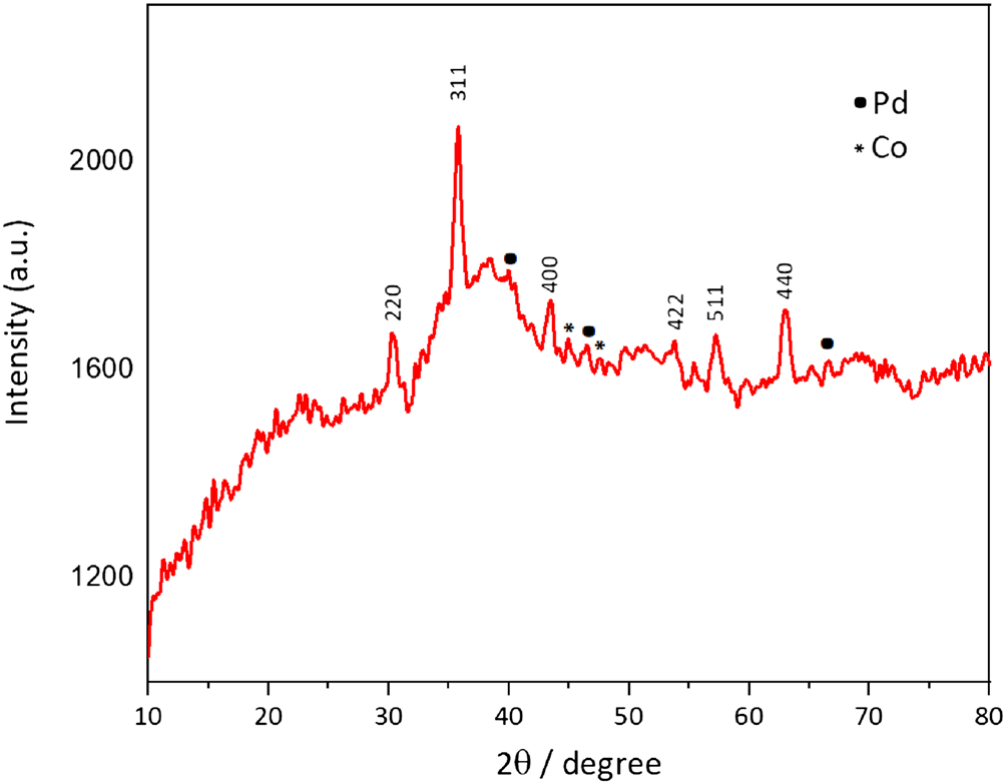
The XRD pattern of γ-Fe_2_O_3_@MBD/Pd-Co.

As shown in Fig. 2, the XRD pattern of γ-Fe_2_O_3_@MBD/Pd-Co established the crystalline structure of the bimetallic Pd-Co alloy nanoparticles. The sample exhibited characteristic diffraction peaks at 2θ = 30.3, 35.7, 43.4, 53.7, 57.2 and 62.9°, which corresponded to the (2 2 0), (3 1 1), (4 0 0), (4 2 2), (5 1 1), and (4 4 0) reflections of the cubic maghemite^29^. The peaks at 2θ values of 40.5, 46.4 and 67.90° are due to the (1 1 1), (2 0 0), and (2 2 0) diffraction peaks corresponding to the face centered cubic Pd (JCPD-46-1043)^10^. The XRD pattern of the sample showed two diffraction signals were placed around 2θ = 44.8 and 47.5° due to the Co species (JCPD-15-0806)^30^. However, those peaks mentioned above slightly shift to larger angles compared with the single metal counterpart^30,31^. The shift could be related to the bimetallic alloy formation.

**Figure 3:**
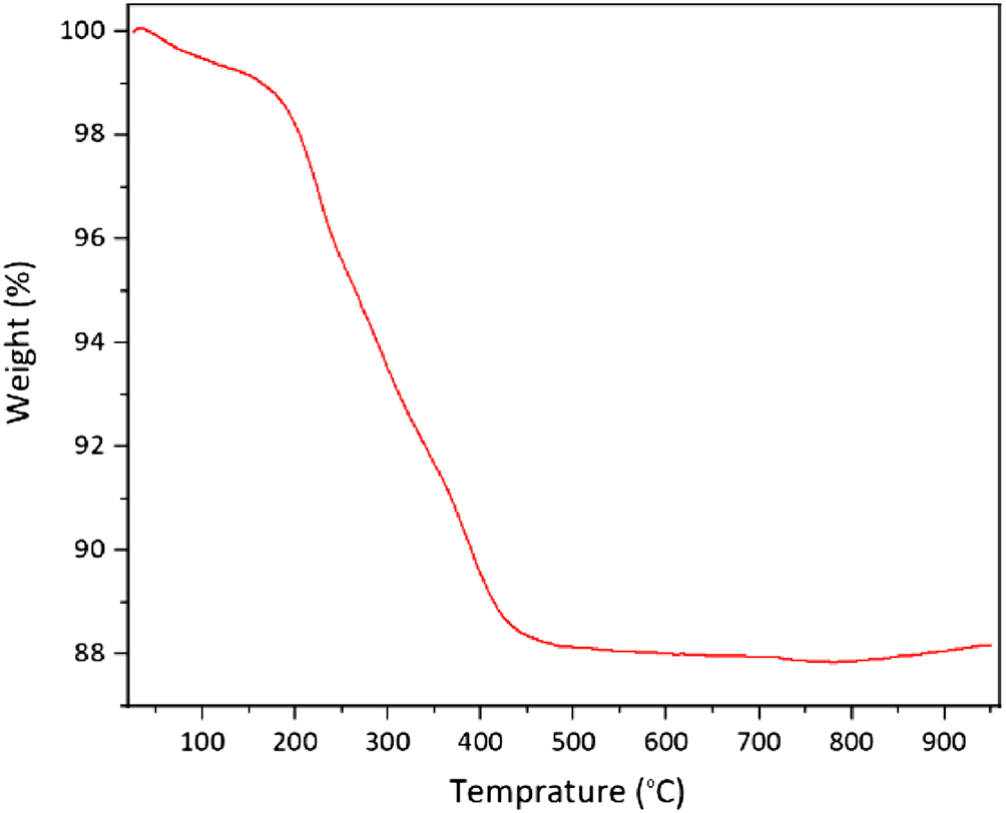
The TG analysis of γ-Fe_2_O_3_@MBD/Pd-Co.

**Figure 4:**
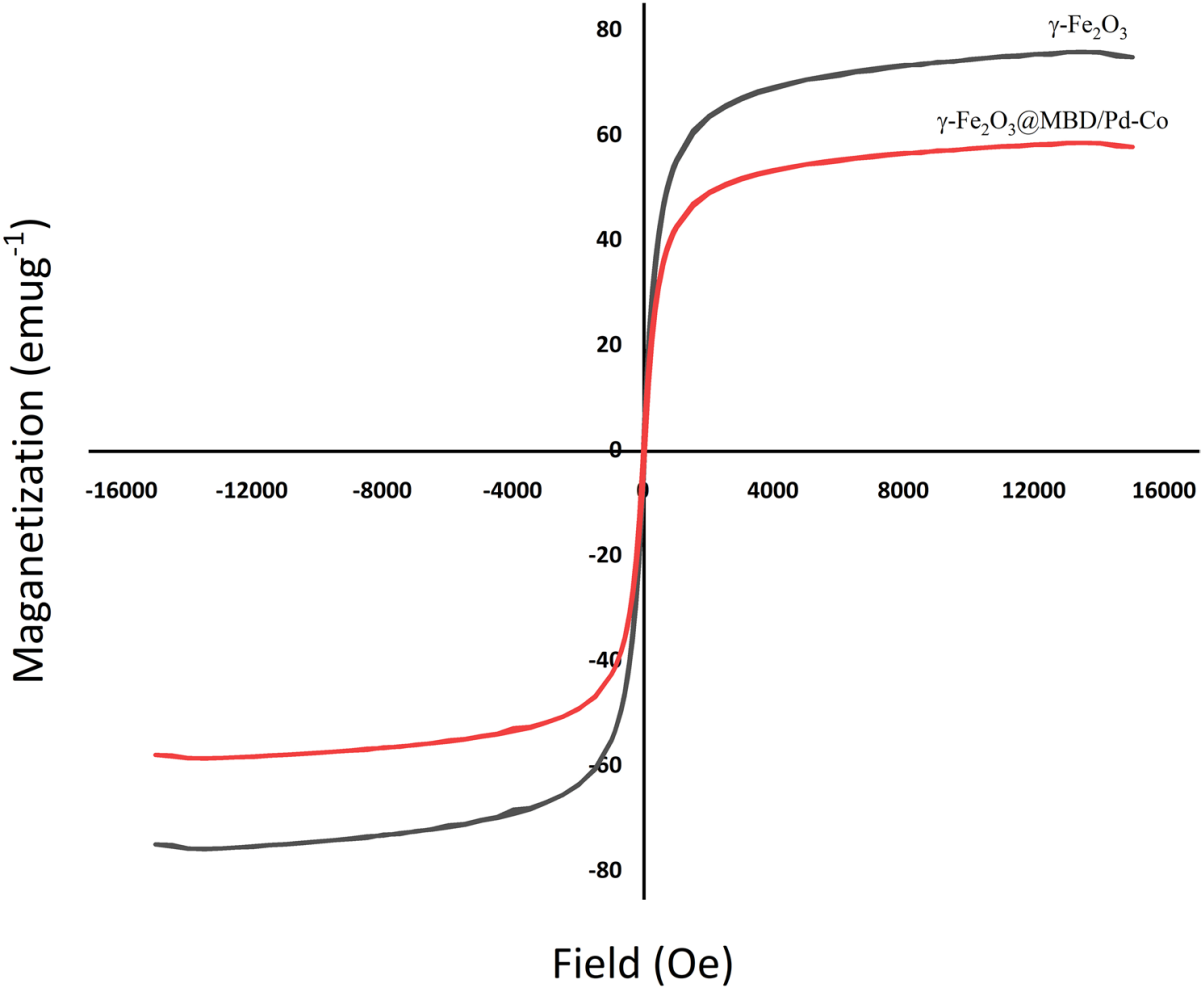
The VSM pattern of γ-Fe_2_O_3_@MBD/Pd-Co and γ-Fe_2_O_3_.

Figure 3 depicts the thermogravimetric analysis of γ-Fe_2_O_3_@MBD. In this plot the first weight loss of 1.18% (< 171 °C), corresponded with the loss of physically adsorbed water. The second weight elimination of 11.61% (171–446 °C) occurred as a consequence of degradation and decomposition of the organic material. These data justified that the melamine-based dendrimers are conveniently grafted on the magnetic nanoparticles. Magnetic properties of γ-Fe_2_O_3_@MBD/Pd-Co and γ-Fe_2_O_3_ were surveyed employing a vibrating sample magnetometer (VSM) at room temperature (Fig. 4). Figure 4 revealed that the saturation magnetization value correspondieng to γ-Fe_2_O_3_@MBD/Pd-Co is approximately 59.07 emu g^−1^. The drop of the saturation magnetization of γ-Fe_2_O_3_@MBD/Pd-Co compared with γ-Fe_2_O_3_ (76.48 emu g^−1^) was related to the coating of γ-Fe_2_O_3_ by melamine-based dendrimer. The magnetization curves did not show a hysteresis loop, which justifiyed the superparamagnetic nature of the resulting NPs. High magnetic properties of NPs were appropriate for their further recovery from the reaction media by the simple magnetic separation using a conventional magnet.

**Figure 5:**
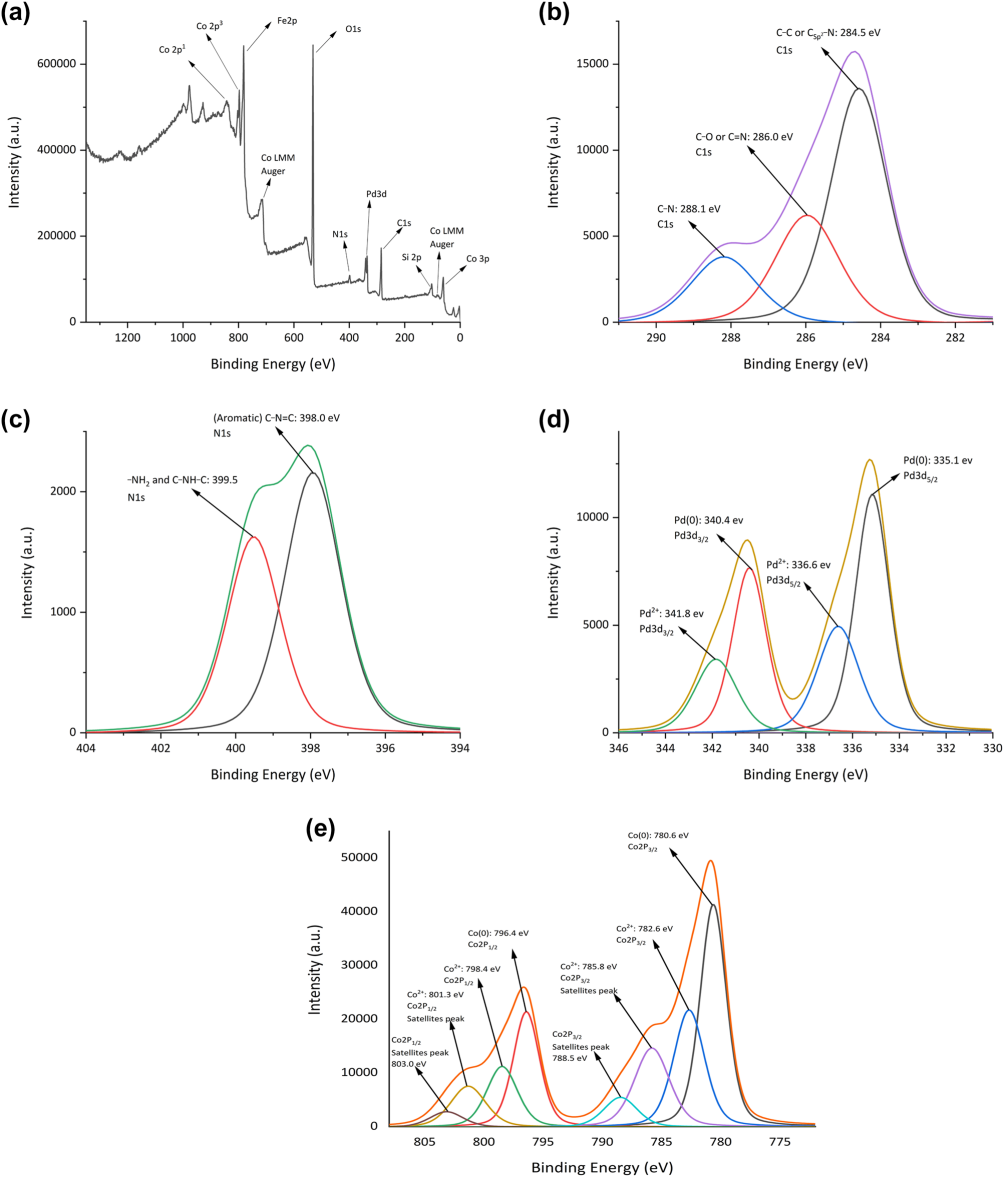
(**a**) XPS analysis of γ-Fe_2_O_3_@MBD/Pd-Co, (**b**) C 1s, (**c**) N 1s, (**d**) Pd 3d and (**e**) Co 2p.

A deep XPS analysis was done to characterize the chemical composition of γ-Fe_2_O_3_@MBD/Pd-Co surface (Fig. 5). The peaks associated to carbon, nitrogen, oxygen, silicon, iron, palladium and cobalt are evidently detected in the XPS plot (Fig. 5a). The C1s spectrum (Fig. 5b) showed binding energies at 284.5 (C_sp2_–N and C–C), 286.0 (C–O and C=N), and 288.1 (C–N) eV^32,33^. Deconvolution of N_1s_ region showed two peaks at 398.0 and 399.5 eV corresponding to C–N=C and N–H, respectively (Fig. 5c)^34^. In Fig. 5d, the peaks at 335.1 (3d_5/2_) and 340.4 eV (3d_3/2_), corresponded to Pd in the zero oxidation state. The peaks at 336.6 (3d_5/2_) and 341.8 eV (3d_3/2_) indicated that a small amount of Pd presents in (II) oxidation state^35,36^. The typical peaks located at 780.6 (2p_3/2_) and 796.4 eV (2p_1/2_) revealed the presence of cobalt (0) in the catalyst (Fig. 5e). It was also detected the presence of both weaker peaks at 782.6 (2p_3/2_) and 798.4 eV (2p_1/2_) corresponding to cobalt (II) species. Different weak satellite peaks at 785.8, 788.6, 801.3 and 803.0 eV^30^, showed the existence of Co_3_O_4_ on the catalyst surface^37^. The displacement of the 2p_3/2_ signal of cobalt to a lower energy and a positive shift in the 3d_5/2_ peak of Pd indicated the alloying of Co with Pd^38^. Moreover, the atomic distribution of palladium *versus* cobalt in the surface of γ-Fe_2_O_3_@MBD/Pd-Co is 1:8.45, which is notably higher than the stoichiometric value (1:4.2) calculated by ICP analysis. This larger value demonstrates that the bimetallic Pd-Co nanoparticles generates a core–shell structure with a cobalt-rich shell and a palladium-rich core^39,40^, and consequently, a larger cobalt surface is exposed.

**Figure 6:**
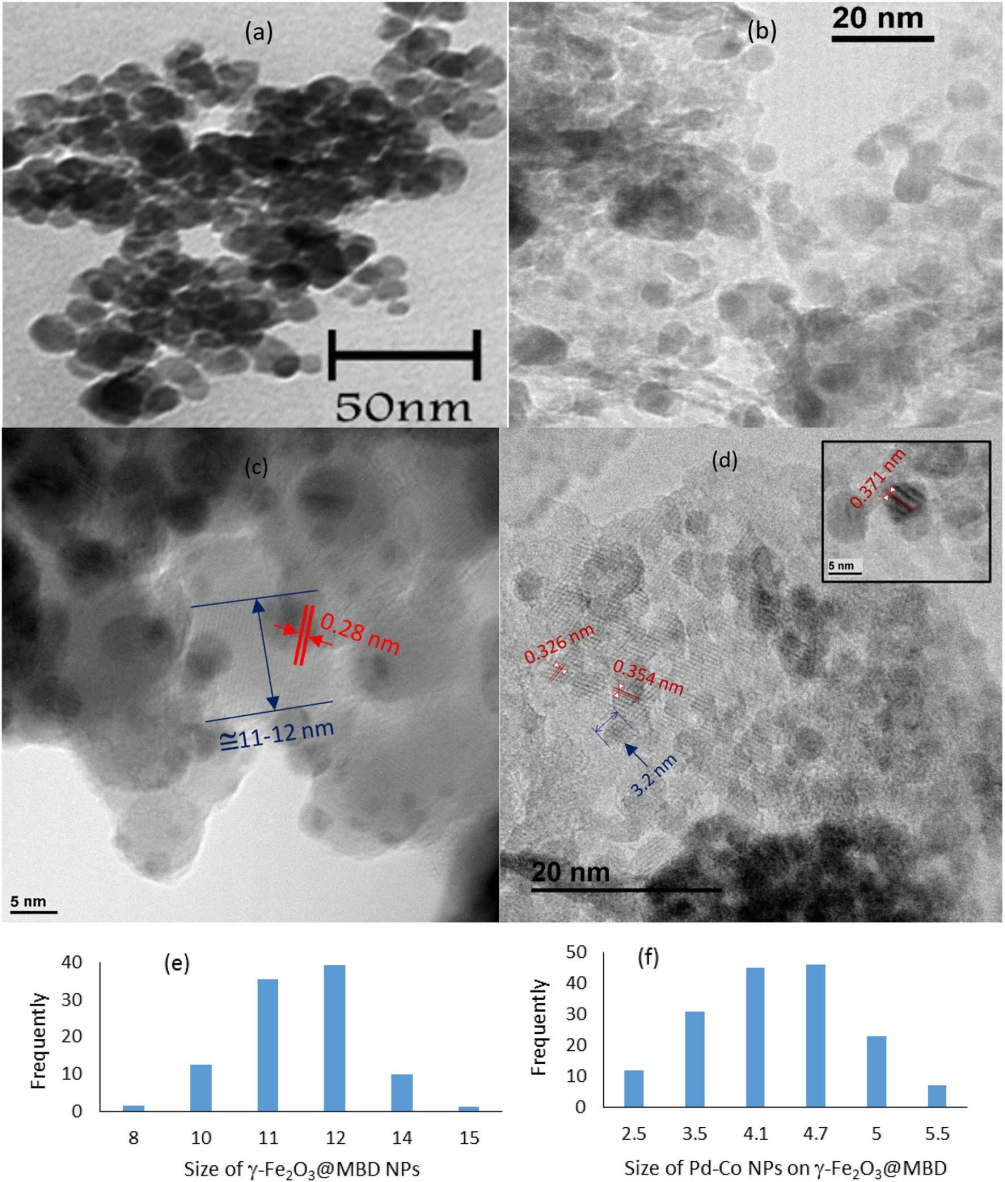
The TEM images of (**a**) γ-Fe_2_O_3_ and (**b**–**d**) γ-Fe_2_O_3_@MBD/Pd-Co and (**e**) the particle size distribution of γ-Fe_2_O_3_@MBD/Pd-Co and (**f**) the particle size distribution of Pd-Co nanoparticles on the γ-Fe_2_O_3_@MBD surface.

The morphology of the surface and size of the particles of the freshly prepared new catalyst were analyzed by transmission electron microscopy (TEM) (Fig. 6). In Fig. 6a a spherical morphology of γ-Fe_2_O_3_ magnetic nanoparticles was shown. Comparing TEM image of γ-Fe_2_O_3_@MBD/Pd-Co (Fig. 6b) with γ-Fe_2_O_3_ showed that dendrimer-magnetite incorporation was dispersed considerably. Figure 6e shows an average diameter size of ~12 nm for γ-Fe_2_O_3_@MBD/Pd-Co. Characteristic lattice fringes for 2 2 0 planes of γ-Fe_2_O_3_ with a d-spacing of 0.28 nm recognized in Fig. 6c. Moreover, TEM images, revealed a homogeneous spreading of the cobalt and palladium alloy nanoparticles immobilized onto the γ-Fe_2_O_3_@MBD surface (Fig. 6c). The size distribution histogram of palladium and cobalt alloy nanoparticles (Fig. 6f), illustrated a high size uniformity of the detected spherical nanoparticles with diameter of about ~ 3–5 nm. Figure 6d depicted the lattice fringe spacing ~ 3.26–3.71 Å related to the Pd-Co alloy nanoparticles^41–43^.

**Figure 7:**
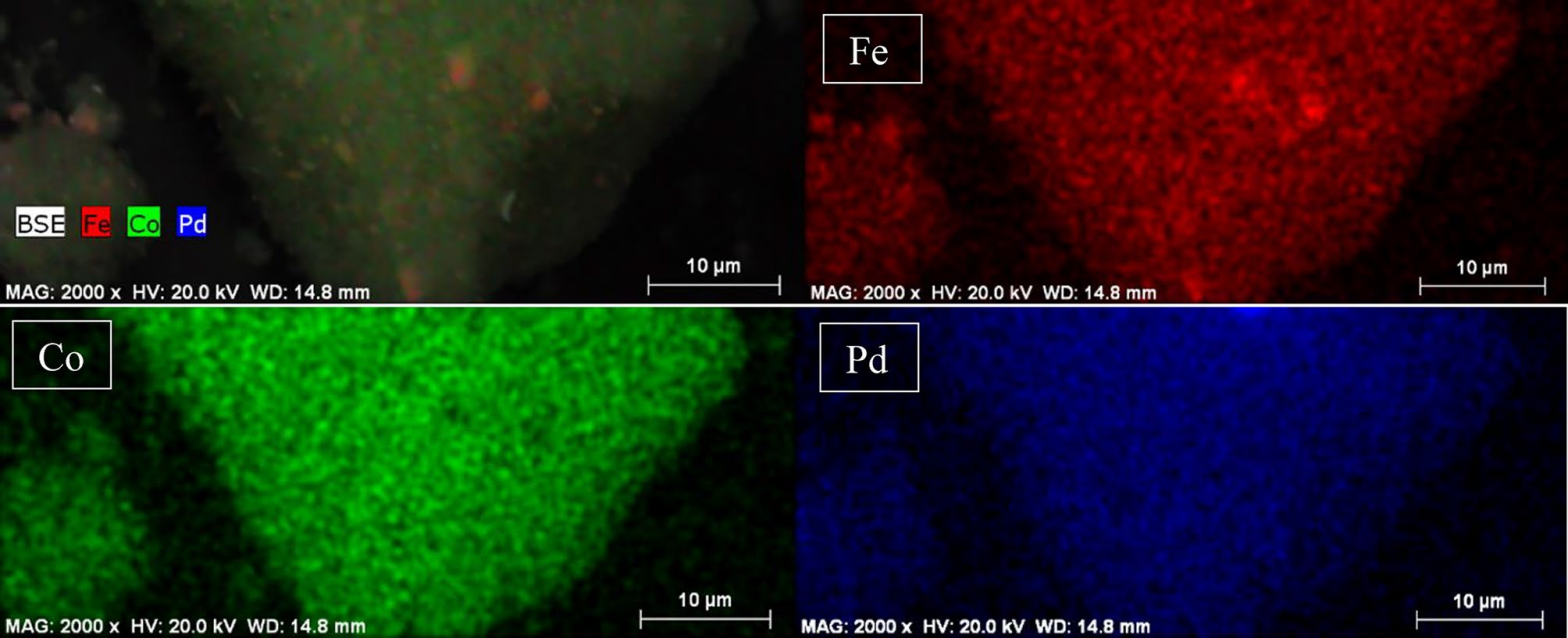
EDS elemental mapping images of the γ-Fe_2_O_3_@MBD/Pd-Co.

The EDS elemental mapping images of the γ-Fe_2_O_3_@MBD/Pd-Co are presented in Fig. 7. The EDS images prove the presence of the Fe, Co and Pd elements on the γ-Fe_2_O_3_@MBD/Pd-Co surface. As it is observed in the Fig. 7, cobalt is denser than palladium on the surface of it.

### Mizoroki–Heck cross-coupling reaction catalyzed by γ-Fe_2_O_3_@MBD/Pd-Co in water

Mizoroki–Heck cross-coupling reactions are applied for the preparation of natural products, pharmaceuticals and biologically active molecules^3,44–46^. These reactions, involving the generation of new carbon–carbon bonds formation, have found several commercial applications for the synthesis of fine chemicals such as herbicide prosulfuron, anti-inflammatory naproxen, or anti-asthma agent Singulair in the multi-ton scale in each year^47^. Generally, the Mizoroki–Heck cross-coupling reaction is catalyzed by palladium complexes^48^. However, another transition metals such as Ni^49^, Co^50^ and Cu^51^ have been recently reported as catalysts for this purpose. Due to the advantageous of bimetallic catalysts such as enhanced catalytic activity which comes from the synergistic effect of the monometallic counterparts, in the past decades, some bimetallic catalysts such as Pd/Cu^52^, Pd/Pt^53^, Pd/Co^11^, Pd/Au^54^, Pd/Ni^55^, Pd/Fe^56^ and etc. have been developed for the Mizoroki–Heck cross-coupling reaction. The synthetic routes published in the literature have certain limitations due to the necessary high temperature, the large amount of catalyst loading, the introduction of additives, the employment of organic solvents and the use of an unrecyclable catalyst. In addition, most of the reported methods suffered from lack of generality for the coupling reactions employing chloroarenes. Continuing with our research on the design and the preparation of novel and attractive catalytic systems to conduct environmentally friendly cross-coupling reactions^4^, herein, we have surveyed the catalytic activity of a novel γ-Fe_2_O_3_@MBD/Pd-Co as the first magnetically recyclable Pd/Co bimetallic catalyst in the Mizoroki–Heck coupling reactions.

At first, the coupling reaction of iodobenzene with *n*-butyl acrylate (1:1.3 molar ratio) in water was investigated as a bench reaction to optimize the effect of the catalyst loading, type of the base and temperature on the reaction completion (Table 1). In the preliminary studies, this model reaction was envestigated in the presence of variable amounts of γ-Fe_2_O_3_@MBD/Pd-Co using Et_3_N as the base (2 equiv.) at 60 °C (Table 1, entries 1–3). The best amount of the catalyst was 0.05 mol% (Table 1, entry 3). The reaction did not work when any amount of the catalyst was not used (Table 1, entry 4). This result proved the crucial role of the catalyst for this transformation. Several bases such as K_2_CO_3_ CsCO_3_, Na_2_CO_3_, NaHCO_3_, KOH and NaOEt were examined for the model reaction using 0.05 mol% of the catalyst at 60 °C (Table 1, entries 5–10). Whitin the bases tested, Et_3_N was found to be the most effective base (Table 1, entry 3). When no bases was used, the corresponding product was obtained with a desirable yield (Table 1, entry 11), which showed that the intrinsic basic sites of melamine-based dendrimer may also promote the reaction in some extent. Subsequently, the reaction was attempted at lower temperatures (Table 1, entries 12 and 13). Here, the product was produced in very low yields and using longer reaction times, especially when the reaction was carried out at room temperature.

**Table 1:** Mizoroki–Heck reaction between iodobenzene and *n*-butyl acrylate catalyzed by γ-Fe_2_O_3_@MBD/Pd-Co using water.


Entry^[a]^	Catalyst (mol%)^[b]^	Base	Time (min)	Isolated yield (%)
1	0.03	Et_3_N	60	91
2	0.04	Et_3_N	60	97
3	0.05	Et_3_N	25	99
4	–	Et_3_N	24 h	Trace
5	0.05	K_2_CO_3_	75	92
6	0.05	CsCO_3_	65	85
7	0.05	Na_2_CO_3_	120	85
8	0.05	NaHCO_3_	120	81
9	0.05	KOH	85	78
10	0.05	NaOEt	30	92
11	0.05	–	70	91
12^[c]^	0.05	Et_3_N	60	93
13^[d]^	0.05	Et_3_N	24 h	65

[a] Reaction conditions: iodobenzene (1 mmol), n-butyl acrylate (1.3 mmol), base (2 mmol), water (1 mL), 60 °C (except for entries 12 and 13), [b] based on the Pd content, [c] 50 °C, [d] room temperature.

The coupling reaction of iodobenzene with *n*-butyl acrylate (1:1.3 molar ratio) under optimized reaction conditions was also studied in the presence of the catalyst containing different ratios of Pd:Co (Table 2). As the ratio of Pd:Co is changed from 1:5.8 to 1:4.2, the efficiency of the catalyst is increased (entry 2). This could be related to increasing the amount of Pd as the most active species in the catalyst. Performing similar reaction in the presence of γ-Fe_2_O_3_@MBD/Pd-Co containing Pd:Co (1:0.8, entry 3) produced the desired product with the same yield as γ-Fe_2_O_3_@MBD/Pd-Co containing Pd:CO (1:4.2). Therefore, the optimum molar ratio of Pd:Co in the catalyst should be 1:4.2.

**Table 2:** Mizoroki–Heck reaction between iodobenzene and n-butyl acrylate catalyzed by γ-Fe_2_O_3_@MBD/Pd-Co containing different ratios of Pd:Co in water.

Entry^[a]^	Pd^[a]^ (mmol g^−1^)	Co^[a]^ (mmol g^−1^)	molar ratio Pd:Co	Pd (mol%)	Time (min)	Isolated yield^[b]^ (%)
1	0.593	3.44	1:5.8	0.04	540	95
2	0.751	3.15	1:4.2	0.05	25	99
3	1.61	1.29	1:0.8	0.1	20	99

[a] Based on ICP-OES analysis, [b] Reaction conditions: iodobenzene (1 mmol), *n*-butyl acrylate (1.3 mmol), base (2 mmol), water (1 mL), 60 °C, catalyst (0.00066 g).

Using the optimized reaction conditions, the scope of Mizoroki–Heck cross-coupling reaction catalyzed by γ-Fe_2_O_3_@MBD/Pd-Co was investigated by employing various aryl halides to react with olefins in water (Table 3). The results of Table 3 reveal that this catalytic protocol is very efficient for running coupling reaction of tested haloarenes. It is worth to mention that any homo-coupling reaction was not occurred in all of the reactions tested. Iodoarenes were coupled with assorted alkenes such as alkyl acrylates and methyl methacrylate following the optimal reaction conditions obtaining the desired products in 95–99% yields (Table 3, entries 1–6). Bromo- and chloroarenes, underwent satisfactory coupling reactions with *n*-butyl acrylate furnishing products in good to high yields (Table 3, entries 7–12). Moreover, a variety of aryl halides underwent successful coupling reaction with styrene under optimized reaction conditions (Table 3, entries 13–19).

**Table 3:**
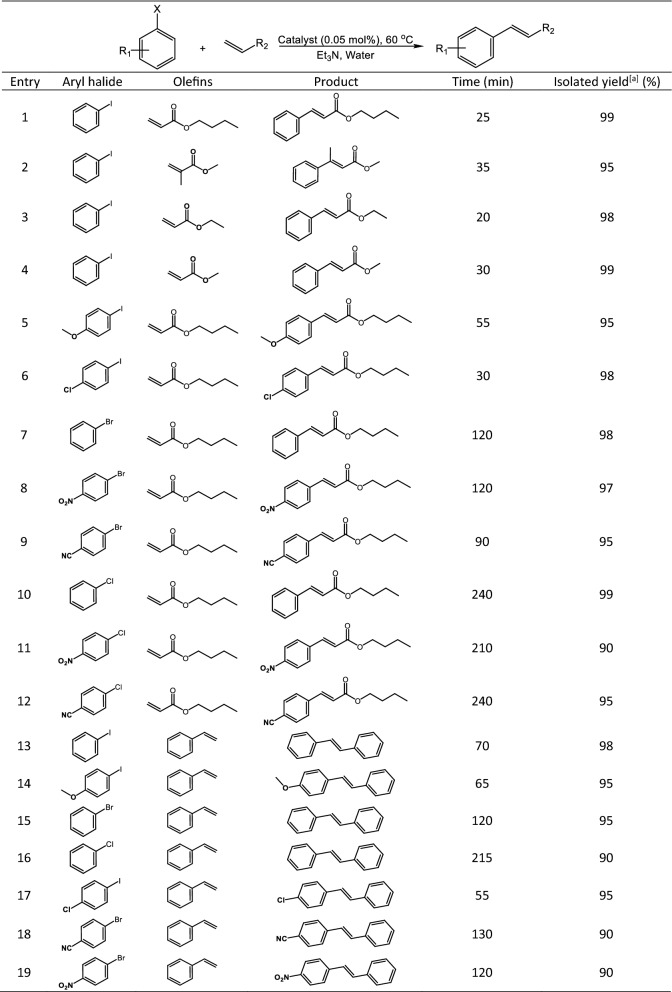
Mizoroki–Heck reaction of various haloarenes with olefins promoted by γ-Fe_2_O_3_@MBD/Pd-Co in aqueous media.

[a] Reaction conditions: catalyst (0.05 mol% based on the Pd content), haloarene (1 mmol), alkene (1.3 mmol), Et_3_N (2 mmol), water (1 mL), 60 °C. Trans-isomer was identified based on ^3^*J*_H-H_ value of 16.0–16.4 Hz for vinylic hydrogens in the ^1^H NMR of the products (Figure S1–S13).

**Figure 8:**
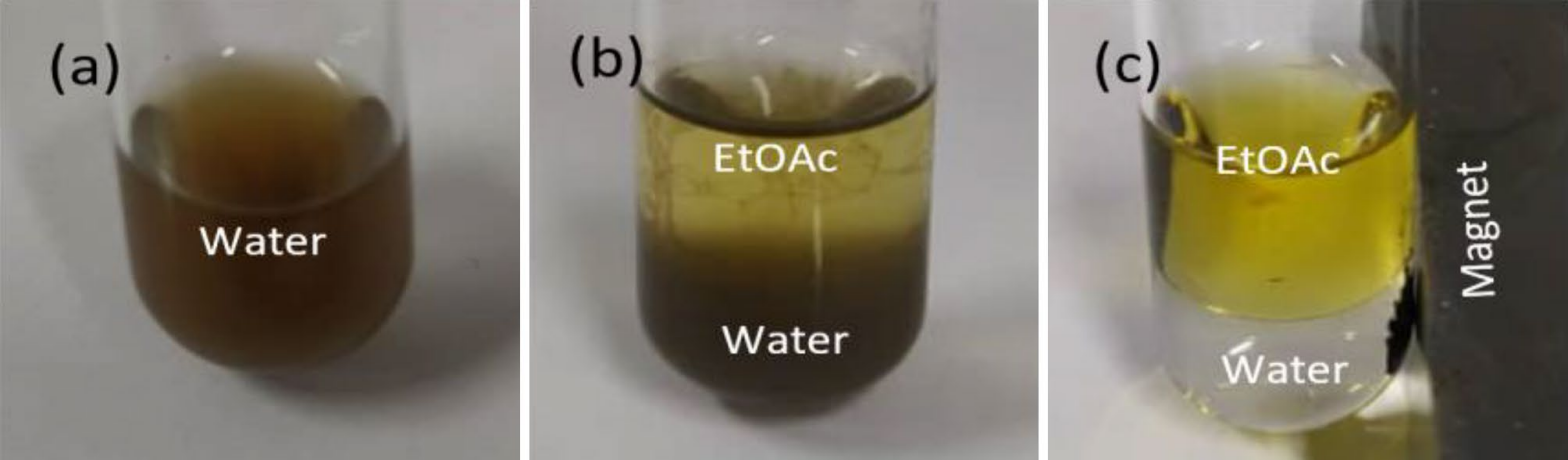
(**a**) Dispersion of γ-Fe_2_O_3_@MBD/Pd-Co in the aqueous media, (**b**) distribution of γ-Fe_2_O_3_@MBD/Pd-Co in water/EtOAc and (**c**) isolation of γ-Fe_2_O_3_@MBD/Pd-Co by an external magnet.

**Figure 9:**
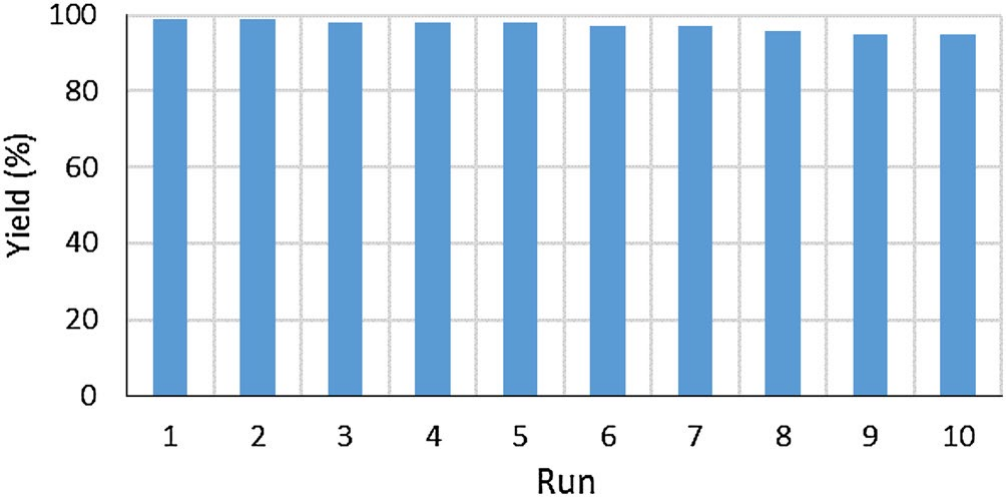
Reusability of γ-Fe_2_O_3_@MBD/Pd-Co in the reaction of n-butyl acrylate (1.3 mmol) with chlorobenzene (1 mmol) in the presence of γ-Fe_2_O_3_@MBD/Pd-Co (0.05 mol% based on the Pd content), base (2 mmol) in water (1 mL) in 4 h.

**Figure 10:**
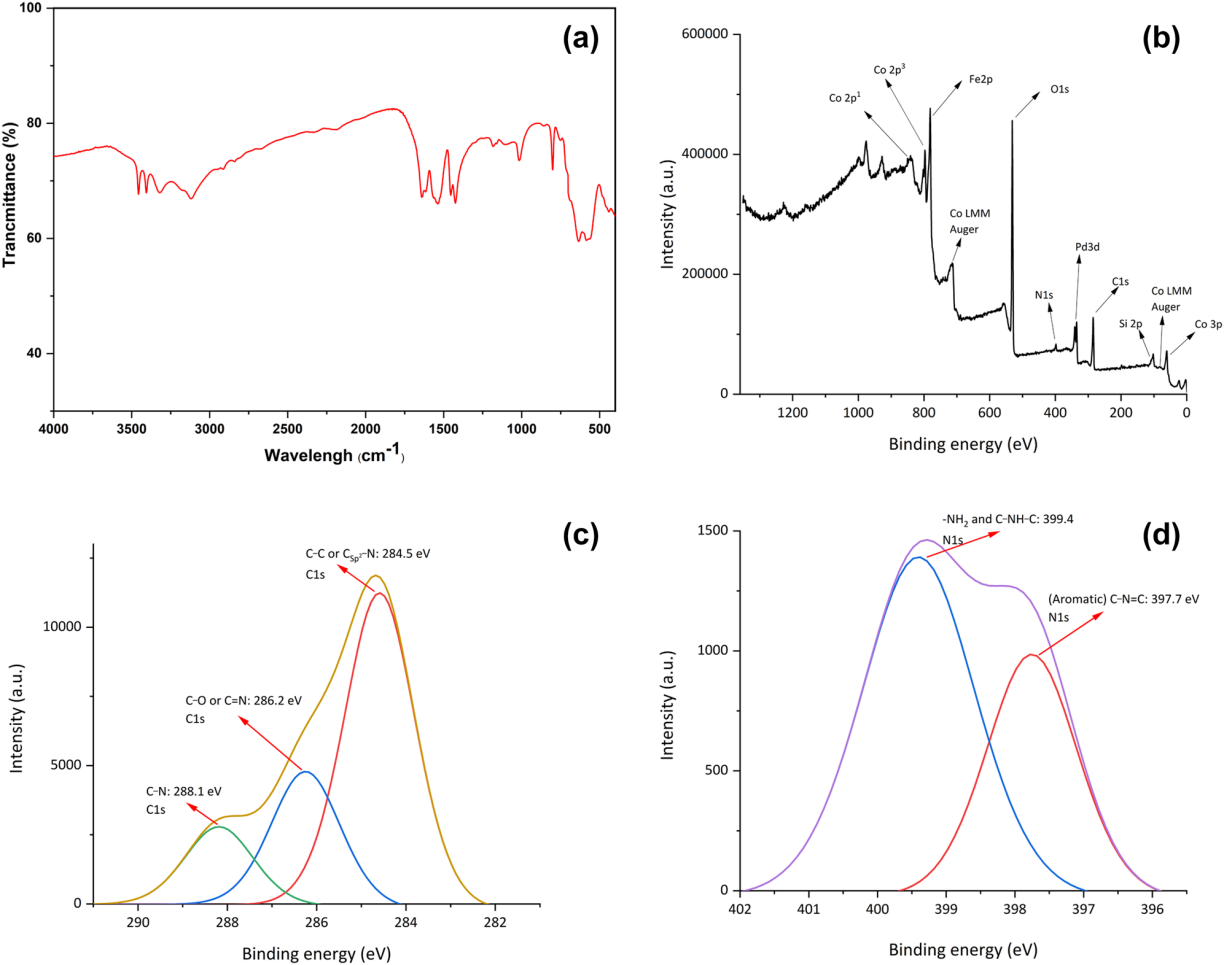

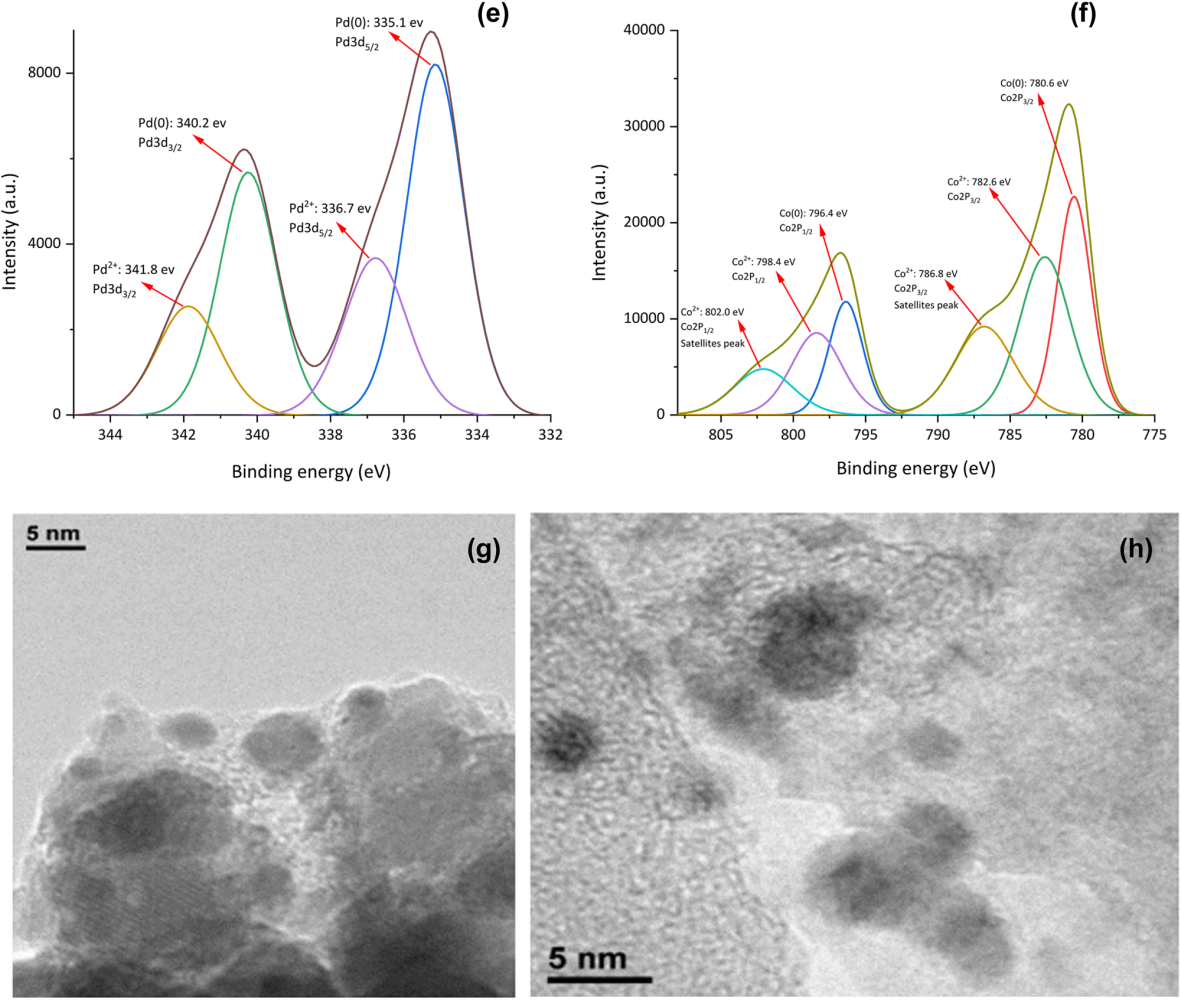
(**a**) FT-IR spectrum, (**b**) XPS pattern, (**c**) C 1s, (**d**) N 1s, (**e**) Pd 3d, (**f**) Co 2p and (**g**,**h**) TEM images of recycled γ-Fe_2_O_3_@MBD/Pd-Co after ten reaction runs.

Since the recovery and recycling of the supported catalysts are very important issues from both the practical and environmental point of view, the reusability of the catalyst in the model reaction was investigated under optimized reaction conditions. Due to the nitrogen and hydroxyl groups in γ-Fe_2_O_3_@MBD/Pd-Co, the catalyst was dissolved in the aqueous layer with no affinity to the organic layer (Fig. 8). According to this feature, the product was extracted with ethyl acetate, while the catalyst remained in the aqueous layer (Fig. 8b). The aqueous layer containing the catalyst was allowed to react with a new batch of iodobenzene, n-butyl acrylate and Et_3_N. At the end, the catalyst was separated from the aqueous phase employing a magnet (Fig. 8c). The catalytic activity of the recovered catalyst was identical to the original one, and the same behavior took place after ten runs (Fig. 9). FT-IR spectrum, XPS pattern and TEM images of the catalyst recovered after the tenth reaction, indicated that the catalyst remained unchanged (Fig. 10).

**Figure 11:**
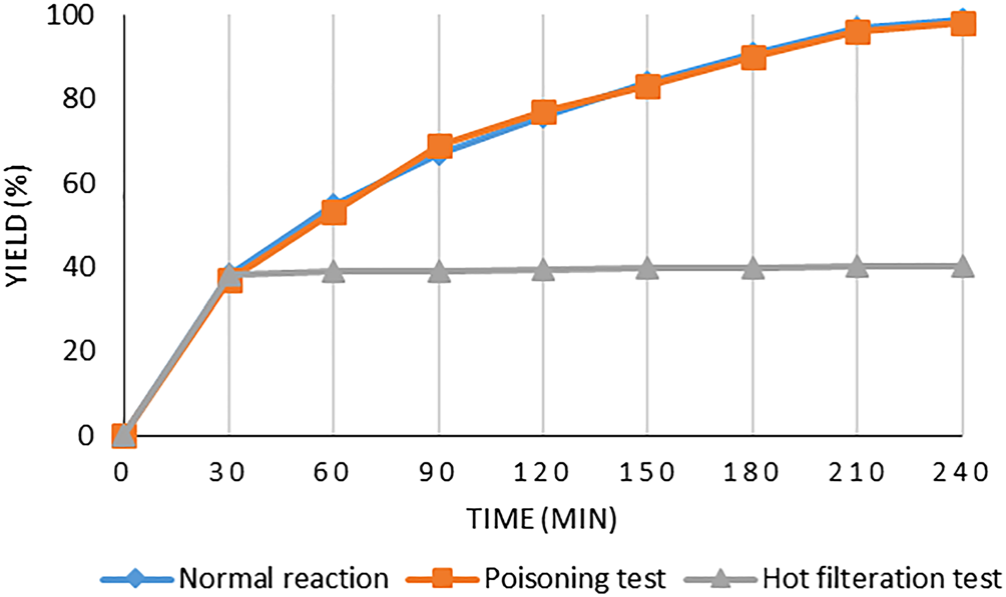
Studies of the heterogeneity of γ-Fe_2_O_3_@MBD/Co-Pd.

The heterogeneous nature of the catalyst was checked by the hot filtration and poisoning tests. In the hot filtration test, after approximate 40% of the coupling reaction of chlorobenzene with *n*-butylacrylate, the solid was separated at the reaction temperature using an external magnetic field and the reaction was permitted to stir for 4 h. Any additional transformation indicated that the catalysis was heterogeneous in nature (Fig. 11). In the poisoning test, S_8_ (0.07 g) was used as a scavenger for the metal. Under this condition, any considerable change in the progress of the reaction was not observed.

### C-N cross-coupling reaction catalyzed by γ-Fe_2_O_3_@MBD/Pd-Co in water

Arylamines and their derivatives possess a paramount importance as intermediates for pharmaceuticals and natural products, agrochemicals, conducting polymers (PANI), and dyes in the chemical industry^57–59^. Arylamines are extremely important ligand for the coordination to transition metals^60^. The Buchwald–Hartwig coupling of amines and aryl halides in the presence of Pd is a great method for the preparation of arylamines^61–63^. Since the discovery of Buchwald–Hartwig amination reaction^64^, research efforts have concentrated on this reaction and significant development have been achieved on the improvement of the conventional reaction conditions such as using diverse transition metals, ligands, and solvents as well as extending substrate scope^65^. The importance of bimetallic catalysts in organic synthesis has recently encouraged organic chemists to use these kinds of catalysts in coupling reactions. Along this line, Fe@Pd nanowire^66^, Pd/Ni nanoparticles^67^ and Pd/Cu complexes^68^ have been used as bimetallic catalysts for the C-N coupling reactions. Encouraged by the facile Mizoroki–Heck cross-coupling reaction catalyzed by γ-Fe_2_O_3_@MBD/Pd-Co, we have tried our catalyst for C–N coupling reaction as well. Surprisingly, to the best of our knowledge, there is not any report on the Buchwald–Hartwig amination reaction catalyzed by Pd/Co bimetallic nanoparticles.

To investigate the catalytic activity of the γ-Fe_2_O_3_@MBD/Pd-Co toward the Buchwald–Hartwig amination reaction, the cross-coupling reaction of chlorobenzene as a poor reactive aryl donor, with aniline in aqueous media, was selected as a bench reaction. Several reaction factors such as the catalyst loading, base and temperature were screened. The results are collected in Table 4. As it is remarked in Table 4, the best yield of the product was observed when 0.07 mol% of the catalyst was used (Table 4, entry 2). Among all the tested organic and inorganic bases (Table 4, entries 6–9), *t*-BuONa was found to be the most effective base. When any bases was not used, the product was isolated in 54% yield after 24 h (Table 4, entry 10), which showed that the intrinsic basic sites of melamine-based dendrimer may also promote the reaction. The model reaction was studied at different temperatures (Table 4, entries 11–13) and the greatest yield of the desired product was produced at 80 °C (Table 4, entry 2). The assessment of the temperature and the catalyst loading was also done for iodobenzene as a highly reactive aryl donor in Table 5. The information given by all these experiments confirmed that 0.05 mol% of the catalyst and 50 °C are the most appropriate conditions to complete the reaction successfully (Table 5, entry 3).

**Table 4:** Buchwald–Hartwig amination reaction of chlorobenzene with aniline catalyzed by γ-Fe_2_O_3_@MBD/Pd-Co under different conditions in water.


Entry^[a]^	Catalyst (mol%)^[b]^	Base	Time(h)	Isolated yield (%)
1	0.1	*t*-BuONa	4	87
2	0.07	*t*-BuONa	5	90
3	–	*t*-BuONa	24	0
4	0.05	*t*-BuONa	10	80
5	0.04	*t*-BuONa	12	75
6	0.07	EtONa	6	83
7	0.07	K_2_CO_3_	24	23
8	0.07	KOH	24	39
9	0.07	Et_3_N	24	56
10	0.07	-	24	54
11^[c]^	0.07	*t*-BuONa	4	90
12^[d]^	0.07	*t*-BuONa	8	81
13^[e]^	0.07	*t*-BuONa	24	Trace

[a] Reaction conditions: chlorobenzene (1 mmol), aniline (1.2 mmol), water (1 mL), 80 °C (except for entries 11–13), [b] based on the Pd content, [c] 100 °C, [d] 70 °C, [e] room temperature.

**Table 5:** Buchwald–Hartwig amination reaction of iodobenzene with aniline catalyzed by γ-Fe_2_O_3_@MBD/Pd-Co under different conditions in water.


Entry^[a]^	Base	T (°C)	Time (h)	Isolated yield (%)
1^[b]^	*t*-BuONa	80	3	88^[c]^
2	*t*-BuONa	80	3	89^[c]^
3	*t*-BuONa	50	4	92
4	*t*-BuONa	40	6	85
5	*t*-BuONa	r.t.	24	70
6^[d]^	*t*-BuONa	50	24	0
7	EtONa	50	5	86
8	K_2_CO_3_	50	24	47
9	KOH	50	24	41
10	Et_3_N	50	24	71

[a] Reaction conditions: iodobenzene (1 mmol), aniline (1.2 mmol), water (1 mL), 0.05 mol% of γ-Fe_2_O_3_@MBD/Pd-Co based on the Pd content (except entry 1 and 6), [b] 0.07 mol% of γ-Fe_2_O_3_@MBD/Pd-Co based on the Pd content, [c] Biaryl as a by-product was also obtained, [d] Without any catalyst.

Next, the chemical scope of this novel bimetallic catalyst was explored and the results are summarized in Table 6. Many aryl iodides were coupled with different arylamines in the presence of γ-Fe_2_O_3_@MBD/Pd-Co and *t*-BuONa under optimized reaction conditions (0.05 mol% of the catalyst, 50 °C) and the corresponding products were obtained in 82–93% yields (Table 6, entries 1–6). The coupling reaction of bromo- and chloroarenes with several arylamines was fruitful using 0.07 mol% of the catalyst at 80 °C (Table 6, entries 7–15).

**Table 6:**
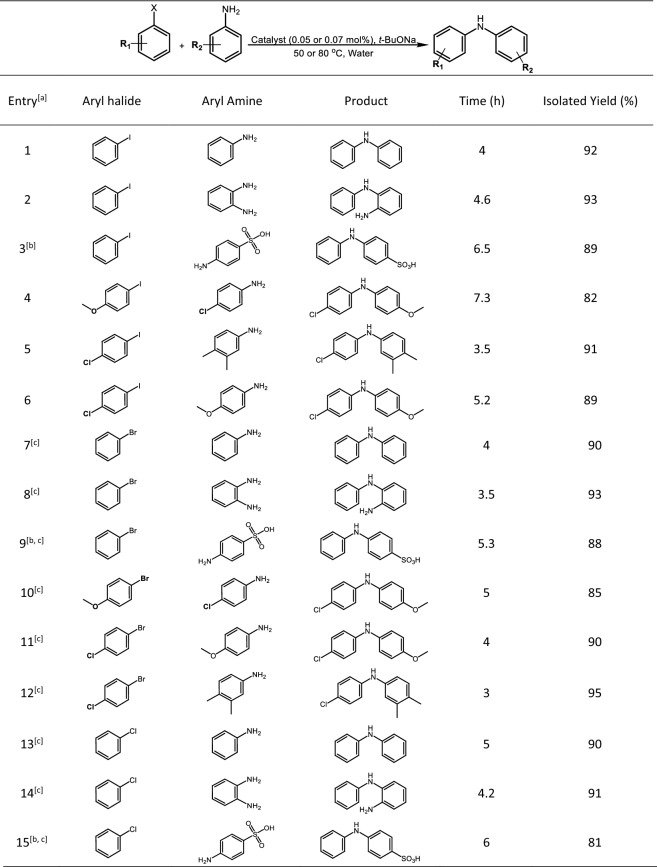
Hartwig–Buchwald amination reaction of assorted haloarenes and arylamines catalyzed by γ-Fe_2_O_3_@MBD/Pd-Co in water.

[a] Reaction conditions: aryl halide (1 mmol), arylamines (1.2 mmol), catalyst (0.05 mol% based on the Pd content, except for entries 7–15), and *t*-BuONa (2 mmol, except for entries 3,9 and 15), 50 °C (except for entries 7–15), The products were characterized by NMR spectroscopy (Figure S14–S18). [b] *t*-BuONa (3 mmol), [c] catalyst (0.07 mol% based on the Pd content), 80 °C.

**Figure 12:**
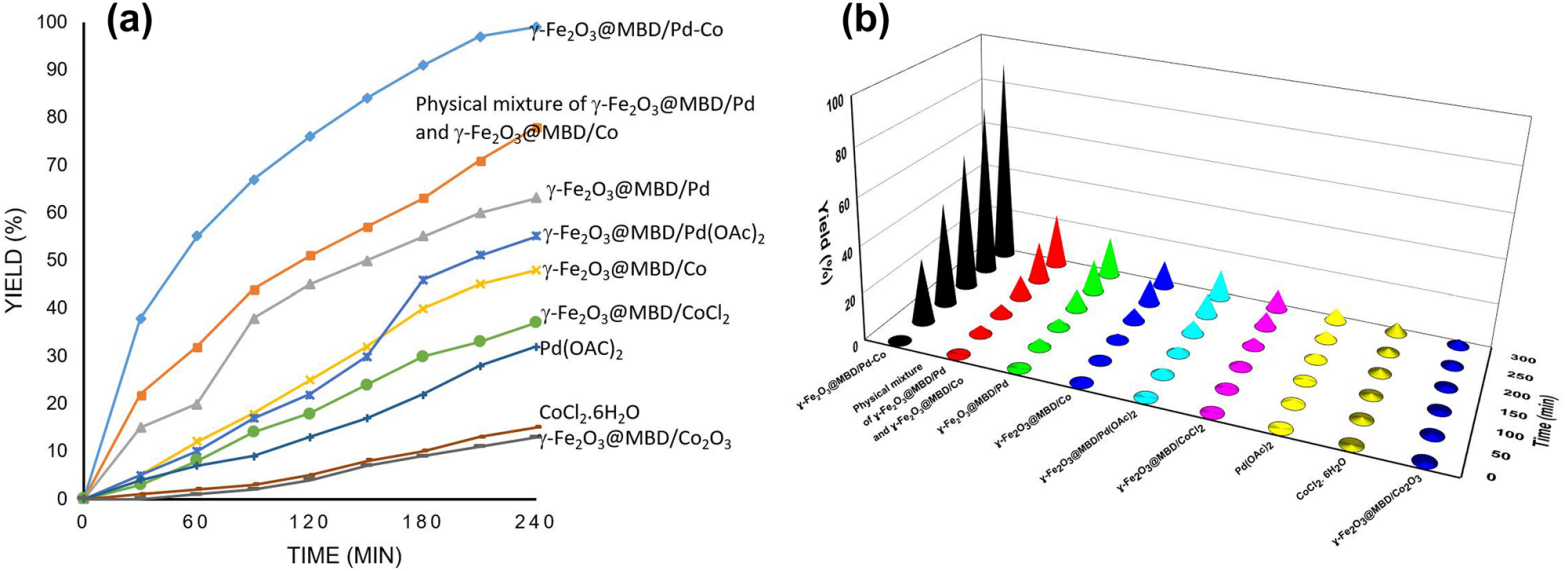
Heck and Buchwald–Hartwig amination reactions of chlorobenzene with *n*-butyl acrylate and aniline, respectively, catalyzed by γ-Fe_2_O_3_@MBD/Pd, γ-Fe_2_O_3_@MBD/Co, Fe_2_O_3_@MBD/Pd(OAc)_2_, γ-Fe_2_O_3_@MBD/CoCl_2_, physical mixture of γ-Fe_2_O_3_@MBD/Pd and γ-Fe_2_O_3_@MBD/Co, Pd(OAc)_2_, CoCl_2_.6H_2_O, and Fe_2_O_3_@MBD/Co_3_O_4_γ-Fe_2_O_3_@MBD/Co-Pd; Reaction conditions: (**a**) chlorobenzene (1 mmol), *n-*butyl acrylate (1.3 mmol), catalyst (0.05 mol% based on the Pd content or 0.21 mol% based on the Co content), Et_3_N (2 mmol), water (1 mL), 50 °C, (**b**) chlorobenzene (1 mmol), aniline (1.2 mmol), water (1 mL), catalyst (0.07 mol% based on the Pd content or 0.29 mol% based on the Co content), *t*-BuONa (2 mmol), water (1 mL), 80 °C.

The Mizoroki–Heck and Buchwald–Hartwig amination reactions of chlorobenzene with n-butyl acrylate and aniline, respectively, catalyzed by monometallic counterparts including γ-Fe_2_O_3_@MBD/Pd, γ-Fe_2_O_3_@MBD/Co, Fe_2_O_3_@MBD/Pd(OAc)_2_, γ-Fe_2_O_3_@MBD/CoCl_2_, physical mixture of γ-Fe_2_O_3_@MBD/Pd and γ-Fe_2_O_3_@MBD/Co, Pd(OAc)_2_, CoCl_2_.6H_2_O, and Fe_2_O_3_@MBD/Co_3_O_4_ were also investigated and the results were compared with the bimetallic catalyst (γ-Fe_2_O_3_@MBD/Pd-Co) (Fig. 12). Obviously, the bimetallic particles provided higher chemical yield (99%) compared with the monometallic counterparts in both reduced and nonreduced forms, physical mixture of monometallic counterparts, and non-supported metal salts and also supported cobalt oxide. The enhanced catalytic activity of these new species is probably originated by a synergistic effect of both metals.

**Scheme 2:**
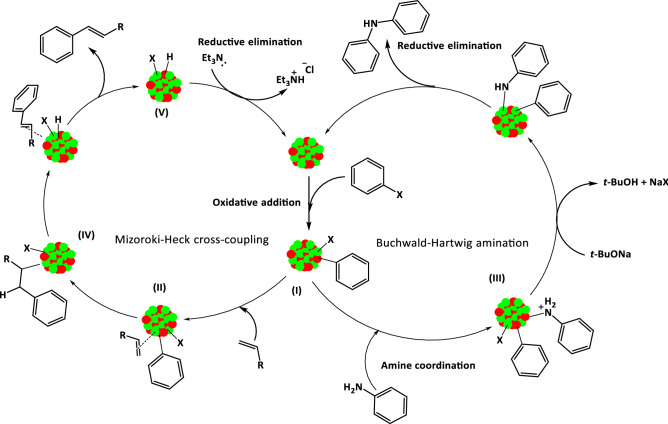
Reasonable mechanisms for the Mizoroki–Heck cross-coupling and Buchwald–Hartwig amination reactions.

Based on the results and fully characterization of the catalyst and other previous contributions^11,69^, we tentatively proposed a plausible mechanisms for Mizoroki–Heck and Buchwald–Hartwig amination reactions in the presence of γ-Fe_2_O_3_@MBD/Pd-Co (Scheme 2). As it is clear in Fig. 12, the Mizoroki–Heck and Buchwald–Hartwig amination reactions proceeded with higher efficiency when involved γ-Fe_2_O_3_@MBD/Pd-Co as a bimetallic catalyst than monometallic counterparts. These finding are in good agreements with a negative shift of the 2p_3/2_ peak of Co and a positive shift in the 3d_5/2_ peak of Pd in the XPS analysis (Fig. 5). The peak shifts indicate that the neighbouring cobalt atoms contribute to increase the electronic density of Pd centres and facilitates the oxidative addition of haloarenes to Pd (0)^11^. Evidently, this step in the coupling reactions is responsible for the higher activity of bimetallic catalysts compared with monometallic counterparts. In the proposed mechanism shown in Scheme 2, at first, the Pd-Co alloy underwent oxidative addition with haloarenes to form an organometallic intermediate** I** on the surface of the catalyst. Then, the reaction proceeds by coordination of olefin or aniline to the intermediate **I** to form intermediate **II** and **III**, respectively. *Syn*-migratory insertion in intermediate **II** generated intermediate complex **IV**, which undergoes *syn-*β-hydride elimination to afford the Mizoroki–Heck coupling product. Finally, the base-assisted elimination of H–X from species **V** occurred to regenerate the catalyst. In the suggested mechanism for Buchwald − Hartwig amination reactions, the product is formed through the base-assisted elimination of H–X from intermediate **III** by subsequent reductive elimination.

Finally, the catalytic activity of γ-Fe_2_O_3_@MBD/Pd-Co was compared with those of reported Pd bimetallic catalysts in the Mizoroki–Heck and Buchwald–Hartwig amination reactions (Table 7). As summarized in Table 7, the most efficiency in the carbon–carbon and carbon–nitrogen coupling reactions of aryl iodides, bromides and chlorides with olefins and aryl amines was observed in the presence of γ-Fe_2_O_3_@MBD/Pd-Co. Most of the reported methods suffer from lack of generality for the coupling reactions of aryl chlorides. Notably, chlorarenes are the most widely available and inexpensive halides compared with other aromatic halides, but are the most challenging ones. Furthermore, the reported procedures have one or more of drawbacks such as requiring high temperature, large quantity of the catalyst, unrecyclable catalysts, additives and organic solvents. High catalytic performance of Fe_2_O_3_@MBD/Pd-Co is the result of the synergetic cooperative effect of both Pd and Co in γ-Fe_2_O_3_@MBD/Pd-Co and its dispersion in water, due to the encapsulation of MNPs by melamine-based dendrimer, which caused better contact between the catalyst and the reactants. Most importantly, the dispersion of the magnetic catalyst facilitates the catalyst recovery and reuse by ten consecutive extraction and at the end magnetic isolation. Among the reported methods, using water as an ecofriendly solvent, simple catalyst recovery and reuse, easy work-up and not needing any additive make our protocol more environmentally benign method for the C–C and C-N cross-coupling reactions.

**Table 7:** Comparison of catalytic activities of our catalyst with various reported bimetallic Pd catalysts for Mizoroki–Heck and Buchwald–Hartwig amination reactions.

Entry^ref^	Coupling reaction	Catalyst: amount of Pd (mol %)	Reaction conditions	Ar-X	Time (h)	Yield (%)
1^70^	Mizoroki–Heck	Pd_63_Sn_37_/C NPs (0.12)	DMF, Et_3_N, 140 °C	I	6	98
				Br		43–95
				Cl		65
2^71^	Mizoroki–Heck	AuPd/Ni-Al-O (0.2)	DMF, K_2_CO_3_, 100 °C	I	8	92
3^54^	Mizoroki–Heck	AuPd/*Euphorbia condylocarpa* M.bieb NPs(0.2)	H_2_O, K_2_CO_3_, 80 °C, CTAB	I	8	89–95
5^72^	Mizoroki–Heck	ZnPd/phenanthroline (1)	DMF, K_3_PO_4_, 120 °C, TEAB	I	24	96
6^73^	Mizoroki–Heck	Ni_0.95_Pd_0.05_ NPs (2)	DMF/H_2_O, K_2_CO_3_, 80 °C	I	9–24	48–76
7^74^	Mizoroki–Heck	Pd_50_Ni_50_/MWCNTs^a^ NPs (0.1)	H_2_O, KOH, 120 °C, TBAB	I	1	99
8^55^	Mizoroki–Heck	PdNi/CL-HA^b^ (0.4)	H_2_O, Bu_3_N, 120 °C, TBAB, N_2_	I	12	100
				Br		96–100
				Cl		28–36
10^8^	Mizoroki–Heck	PdCu/AOT^c^/*iso*-octane NPs (0.1)	DMF, Et_3_N, 100 °C	I	18	93–100
				Br		74–97
				Cl		66–85
11^75^	Mizoroki–Heck	Pd_0.1_Au_0.16_/Fe_3_O_4_@LDH^d^ (0.05)	DMF/H_2_O, K_2_CO_3_, 120 °C	I	0.67–2	89–99
				Br	2.5–24	43–98
12^76^	Mizoroki–Heck	Pd-M(M = Ag, Ni, and Cu)/C NPs (0.3)	Acetonitrile, Et_3_N, 82 °C	I	3	35–62
13^77^	Mizoroki–Heck	MnPd@Py-2,2′-BPyPh COF (0.08)	Acetonitrile, K_2_CO_3_, 80 °C, N_2_	I	24	95
14^78^	Mizoroki–Heck	Hierarchical Pd–Ni/Ni(OH)_2_ (0.02)	TBAA, Et_3_N, 160 °C	I	24	87
15^11^	Mizoroki–Heck	Pd-Co/CoAl-LDH (0.02)	DMF/H_2_O, K_2_CO_3_, 120–140 °C	I	0.5–1.5	91–100
				Br	4–5	92–93
				Cl	10	64
16^This work^	Mizoroki–Heck	γ-Fe_2_O_3_@MBD/Pd-Co NPs (0.05)	H_2_O, Et_3_N, 60 °C	I	0.4–1.1	95–99
				Br	1.5–2	95–98
				Cl	4–5	90–99
17^79^	Buchwald–Hartwig amination	PdAu/C (3)	DMSO, *t*-BuOK, 100 °C	Cl	12	80–99
18^67^	Buchwald–Hartwig amination	Pd–Ni/B-NPs (0.45)	K_2_CO_3_, H_2_O, 80 °C	Br	5–8	86–100
				Cl	8	69–100
19^This work^	Buchwald–Hartwig amination	γ-Fe_2_O_3_@MBD/Pd-Co NPs (0.05–0.07)	*t*-BuONa, H_2_O, 50–80 °C	I	3.5–7.3	89–93
				Br	3.5–5	88–93
				Cl	4.2–6	81–91

[a] Multi-walled carbon nanotube, [b] humic acids, [c] aerosol-OT, [d] layered double hydroxide.

## Conclusion

In summary, in this work, melamine-based dendrimer supported on γ-Fe_2_O_3_ magnetic nanoparticles was efficiently employed as a suitable material for the in situ preparation of palladium-cobalt nanoparticles by reduction with NaBH_4_. TEM images indicated uniform distribution of fairly small palladium and cobalt alloy nanoparticles supported on the surface of the catalyst. The catalyst was characterized by FT-IR, XRD, XPS, VSM, TGA, ICP and elemental analysis. It was used as a new water-dispersible/magnetically recyclable Pd/Co heterogeneous bimetallic catalyst (γ-Fe_2_O_3_@MBD/Pd-Co) for the efficient Mizoroki–Heck and Buchwald–Hartwig amination reactions in water. A variety of iodo-, bromo- and chloroarenes successfully reacted with acrylates, styrene and anilines to yield the corresponding products. Using this protocol, products were achieved in good to high yields in water as an ecofriendly solvent and without using any additives. The synergistic cooperative effect of both Pd and Co in the encapsulated catalyst leads to high catalytic performance in the cross-coupling reactions in aqueous media. The dispersion of the magnetic catalyst facilitates the catalyst recovery and reuse by ten consecutive extraction and at the end magnetic isolation. The experiments based on isolation of the catalyst in the hot filtration test and using S_8_ in the poisoning test, showed that the observed catalysis was heterogeneous in nature. Use of water as an ecofriendly solvent, simple catalyst recovery and reuse, ease of work-up and not needing any additive make this method an environmentally benign procedure for the carbon–carbon and carbon–nitrogen cross-coupling reactions (Supplementary Information).

## Supplementary Information

Supplementary Information.
