# Supplementary material for: A new nanomagnetic Pd-Co bimetallic alloy as catalyst in the Mizoroki–Heck and Buchwald–Hartwig amination reactions in aqueous media
Source: Sci Rep. 2021 Aug 23;11:17025. doi: 10.1038/s41598-021-95931-6 (PMC8382703; doi:10.1038/s41598-021-95931-6)
Supplement: Supplementary file 1 — Supplementary Information. [file 41598_2021_95931_MOESM1_ESM.pdf]

# A new nanomagnetic Pd-Co bimetallic alloy as catalyst in the Mizoroki-Heck and Buchwald–Hartwig amination reactions in aqueous media

Sara Sobhani,[1]* Hamed Zarei,[1] José Miguel Sansano[2]

[1]Department of Chemistry, College of Sciences, University of Birjand, Birjand, Iran, email: ssobhani@birjand.ac.ir, sobhanisara@yahoo.com.

[2]Departamento de Química Orgánica, Facultad de Ciencias, Centro de Innovación en Química Avanzada (ORFEO-CINQA) and Instituto de Síntesis Orgánica (ISO), Universidad de Alicante, Apdo. 99, 03080-Alicante, Spain.

## General information

Chemicals were purchased from Merck Chemical Company. NMR spectra were recorded on a Bruker Avance DPX-400 and 300 using deutrated $CDCl_3$ and DMSO-$d_6$ as solvent and TMS as internal standard.

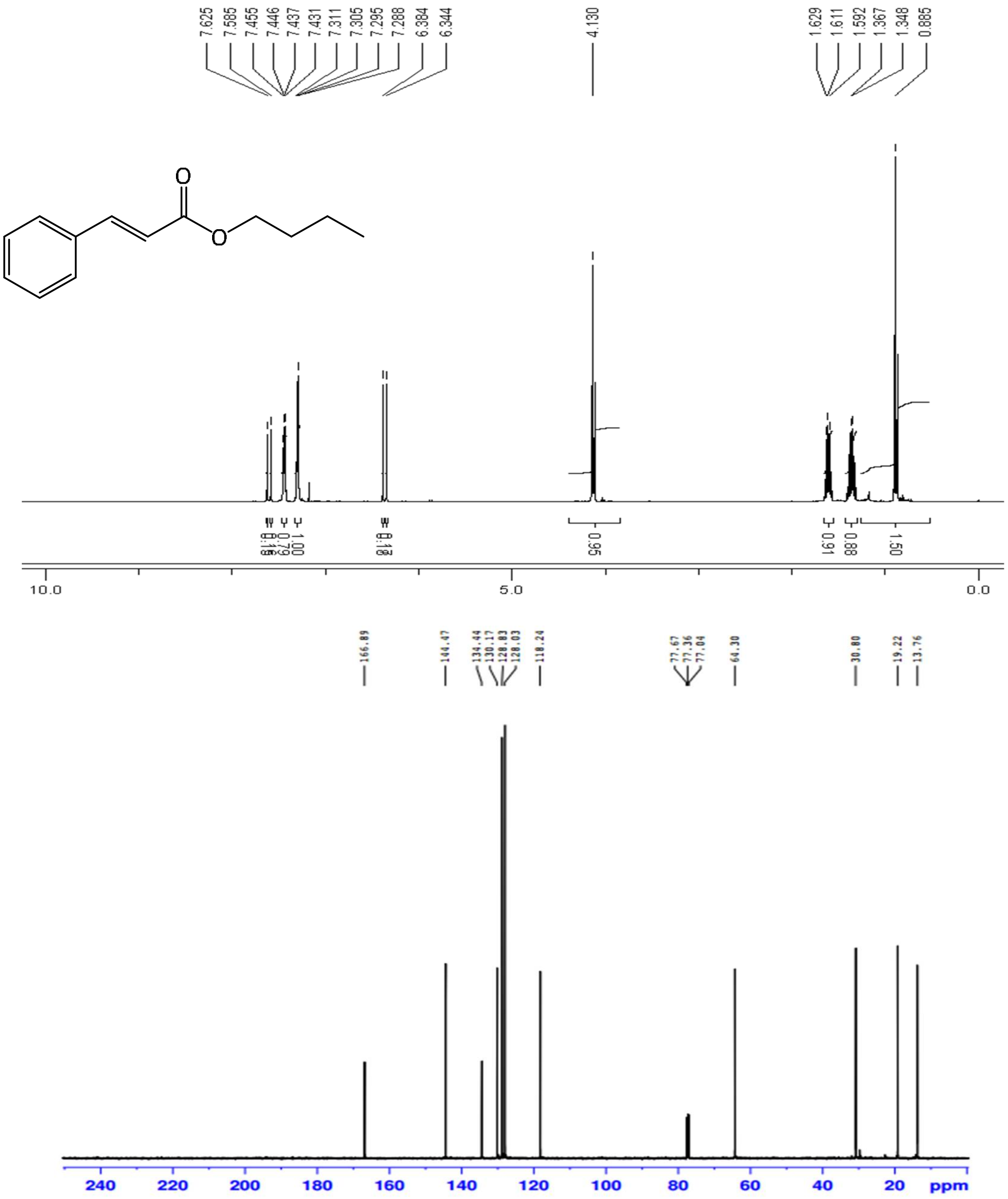


**Figure S1:** $^{1}$H NMR and $^{13}$C NMR spectra of (E)-*n*-butyl cinnamate

$^{1}$H NMR (400 MHz, $CDCl_3$): $\delta$ 7.60 (d, 1H, $^{3}J$ = 16.4 Hz), 7.43-7.45 (m, 2H), 7.29-7.30 (m, 3H), 6.36 (d, 1H, $^{3}J$ = 16.0 Hz), 4.13 (t, 2H, $^{3}J$ = 6.8 Hz), 1.59-1.62 (m, 2H), 1.34-1.36 (m, 2H), $\delta$ 0.88 (t, 3H, $^{3}J$ = 7.6 Hz) ppm. $^{13}$C NMR (100 MHz, $CDCl_3$), $\delta$ 166.8, 144.4, 134.4, 130.1, 128.8, 128.0, 118.2, 64.3, 30.8, 19.2, 13.7 ppm.

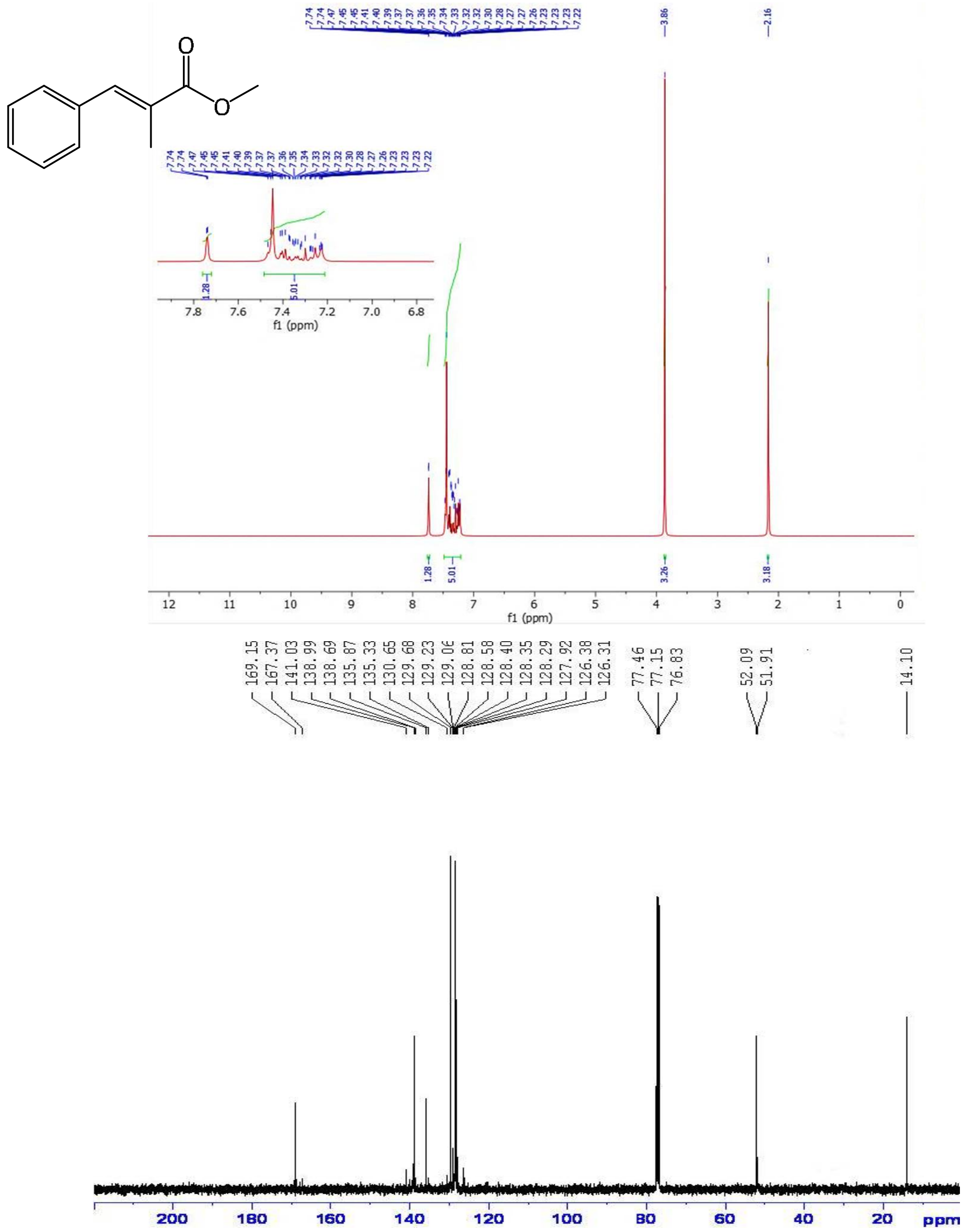


**Figure S2:** $^{1}H$ NMR and $^{13}C$ NMR spectra of (E)-methyl 2-methyl-3-phenylacrylate

$^{1}H$ NMR (300 MHz, $CDCl_3$): δ 7.74 (s, 1H), 7.22-7.47 (m, 5H), 3.86 (s, 3H), 2.16 (s, 3H) ppm.$^{13}C$ NMR (100 MHz, $CDCl_3$), δ 169.1, 138.9, 135.8, 129.6, 127.9, 52.0, 14.1 ppm.

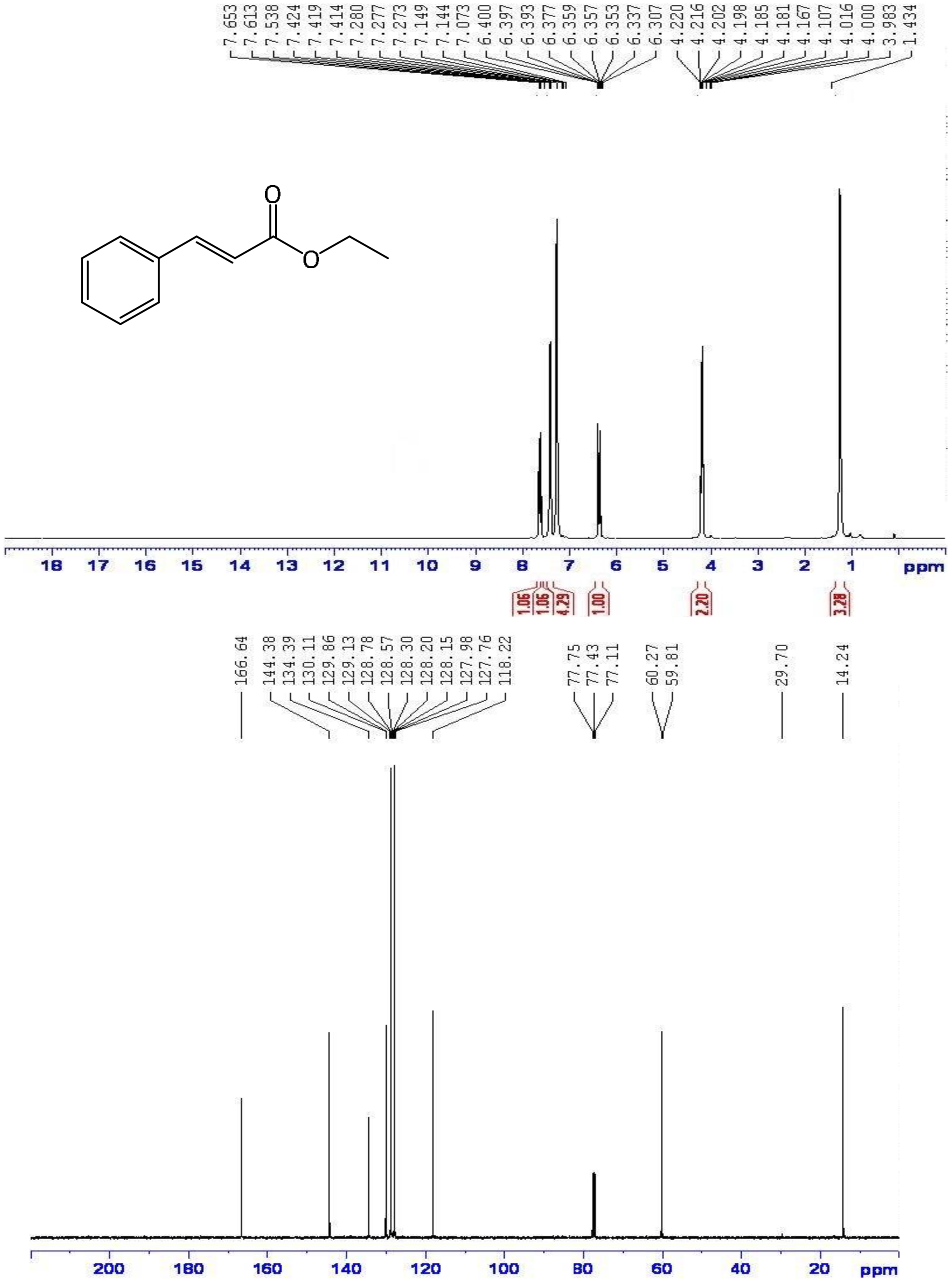


**Figure S3:** $^{1}$H NMR and $^{13}$C NMR spectra of (E)-ethyl cinnamate

$^{1}$H NMR (400 MHz, $CDCl_3$): δ 7.63 (d, 1H, $^{3}J$ = 16.0 Hz), 7.41-7.42 (m, 2H), 7.27-7.28 (m, 3H), 6.35 (d, 1H, $^{3}J$ = 16.0 Hz), 4.19 (q, 2H, $^{3}J$ = 8.0 Hz), 1.43 (t, 3H, $^{3}J$ = 8.0 Hz) ppm.$^{13}$C NMR (100 MHz, $CDCl_3$), δ 166.6, 144.3, 134.3, 130.1, 128.7, 127.9, 11.2, 60.0, 14.2 ppm.

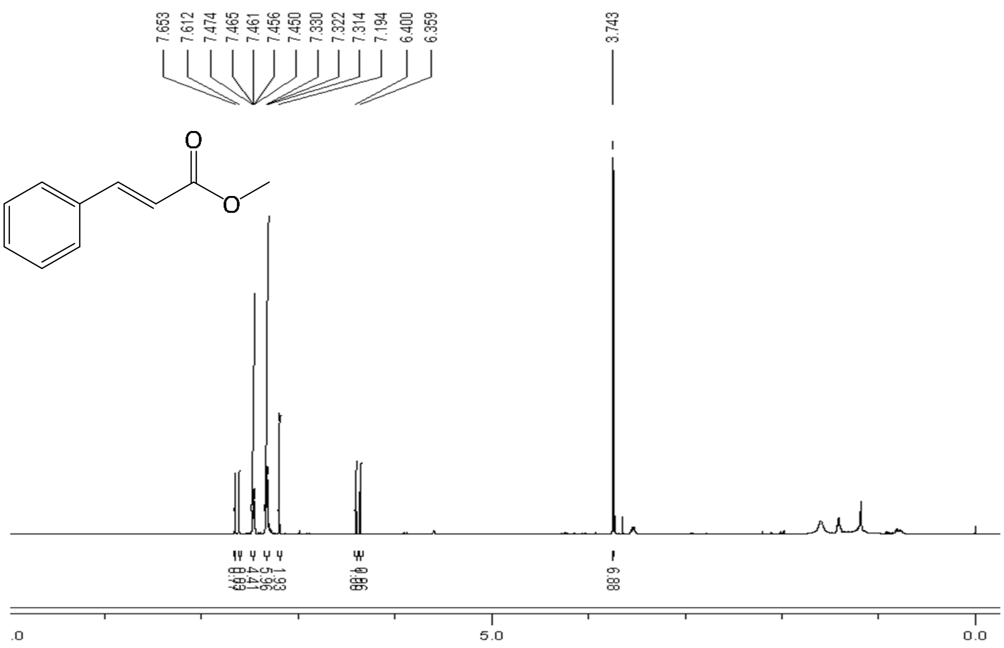

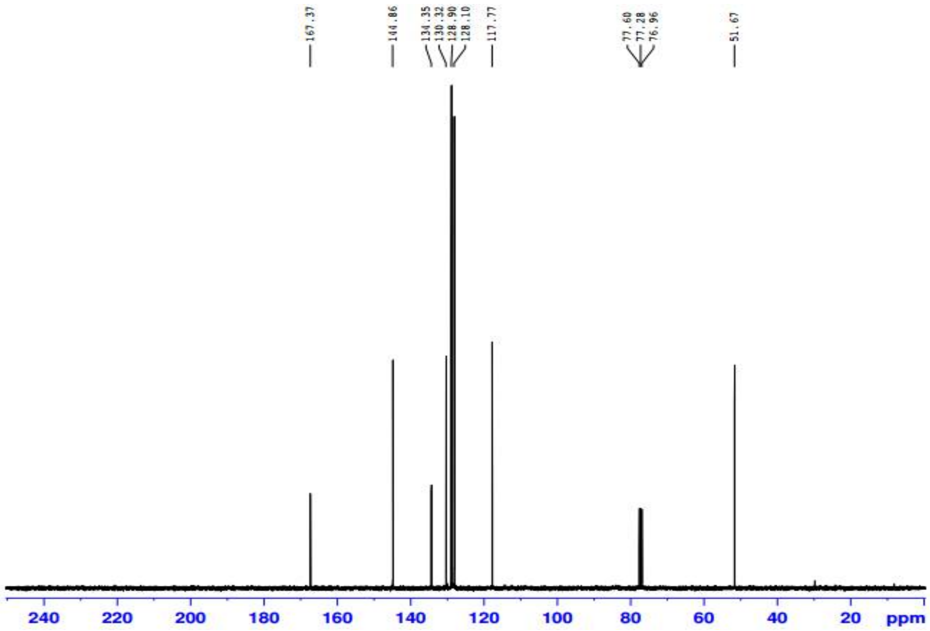

**Figure S4:** $^{1}H$ NMR and $^{13}C$ NMR spectra of (E)-methyl cinnamate

$^{1}H$ NMR (400 MHz, $CDCl_3$): δ 7.63 (d, 1H, $^{3}J$ = 16.4 Hz), 7.45-7.47 (m, 2H), 7.31-7.33 (m, 3H), 6.38 (d, 1H, $^{3}J$ = 16.4 Hz), 3.74 (s, 3H) ppm. $^{13}C$ NMR (100 MHz, $CDCl_3$), δ 167.3, 144.8, 134.3, 130.3, 128.9, 128.1, 117.7, 51.6 ppm.

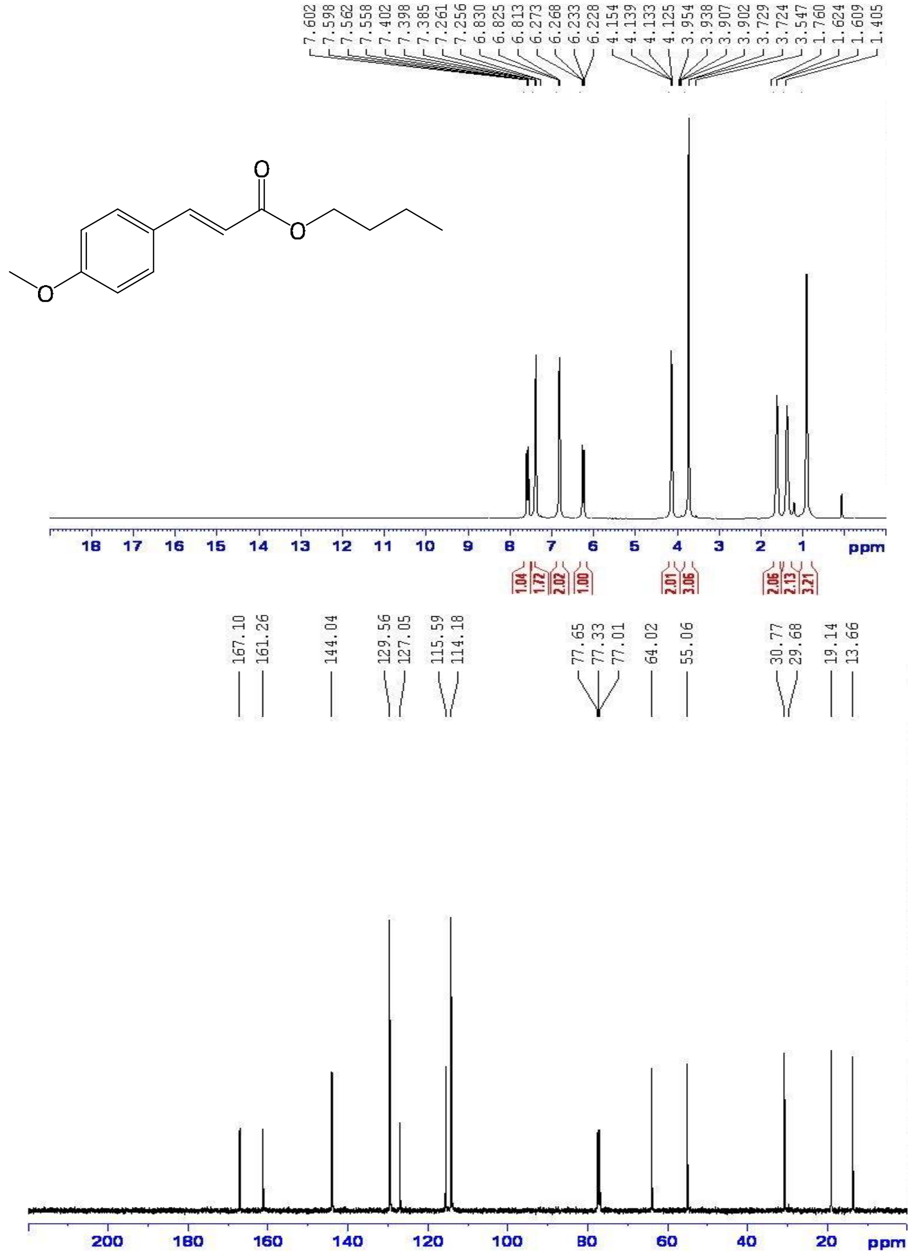


**Figure S5:** $^{1}$H NMR and $^{13}$C NMR spectra of (E)-*n*-butyl 3-(4-methoxyphenyl) acrylate

$^{1}$H NMR (400 MHz, $CDCl_3$): $\delta$ 7.58 (d, 1H, $^{3}J$ = 16.0 Hz), 7.39 (d, 3H, $^{3}J$ =4.2 Hz), 6.24 (d, 2H, $^{3}J$ = 16.0 Hz), 4.13 (m, 2H, $^{3}J$ =7.0 Hz), 3.72 (s, 3H), 1.60-1.62 (m, 2H), 1.35-1.40 (m, 2H), 0.91 (t, 3H, $^{3}J$ =7.0 Hz) ppm.$^{13}$C NMR (100 MHz, $CDCl_3$), δ 167.1, 161.2, 144.0, 129.5, 127.0, 115.5, 114.1, 64.0, 55.0, 30.7, 19.1, 13.6 ppm.

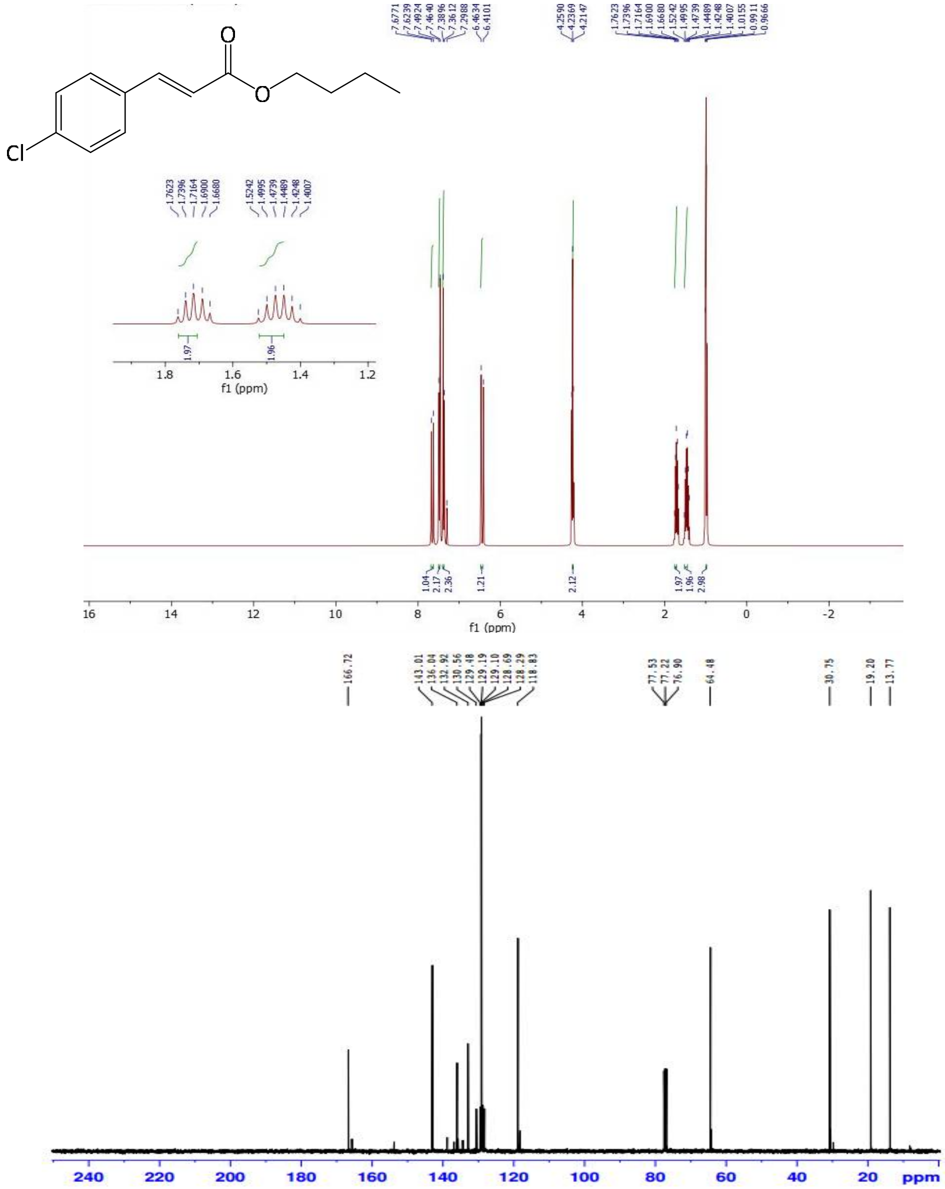


**Figure S6:** $^{1}H$ NMR and $^{13}C$ NMR spectra of (E)-*n*-butyl 3-(4-chlorophenyl) acrylate

$^{1}H$ NMR (300 MHz, $CDCl_3$): $\delta$ 7.65 (d, 1H, $^{3}J$ = 16.0 Hz), 7.47 (d, 2H, $^{3}J$ = 8.5 Hz), 7.37 (d, 2H, $^{3}J$ = 8.5 Hz), 6.43 (d, 1H, $^{3}J$ = 16.0 Hz), 4.24 (t, 2H, $^{3}J$ = 6.6 Hz), 1.40-1.52 (m, 2H), 1.67-1.76 (m, 2H), 0.99 (t, 3H, $^{3}J$ = 7.3 Hz) ppm. $^{13}C$ NMR (100 MHz, $CDCl_3$), $\delta$ 166.7, 143.0, 136.0, 132.9, 129.4, 129.1, 118.8, 64.4, 30.7, 19.2, 13.7 ppm.

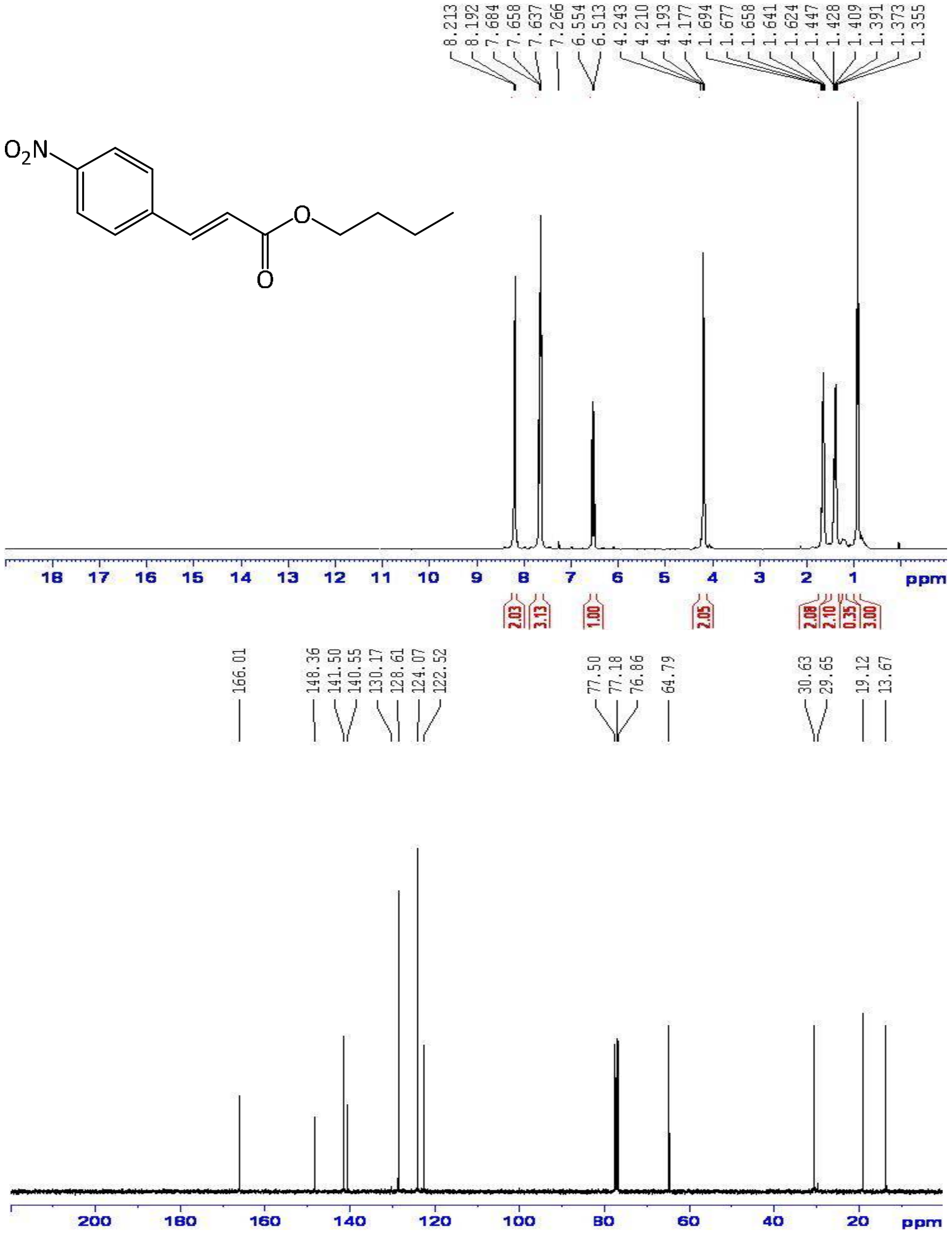


**Figure S7:** $^1$H NMR and $^{13}$C NMR spectra of (E)-*n*-butyl 3-(4-nitrophenyl) acrylate

$^1$H NMR (400 MHz, $CDCl_3$): $\delta$ 8.20 (d, 2H, $^3J$ = 8.0 Hz), 7.63-7.68 (m, 3H), 6.53 (d, 1H, $^3J$ = 16.0 Hz), 4.17-4.24 (m, 2H), 1.62-1.69 (m, 2H), 1.35-1.44 (m, 2H), 0.92 (t, 3H, $^3J$ = 7.2 Hz) ppm. $^{13}$C NMR (100 MHz, $CDCl_3$), δ 166.0, 148.3, 141.5, 140.5, 128.6, 124.0, 122.5, 64.7, 30.6, 19.1, 13.6 ppm.

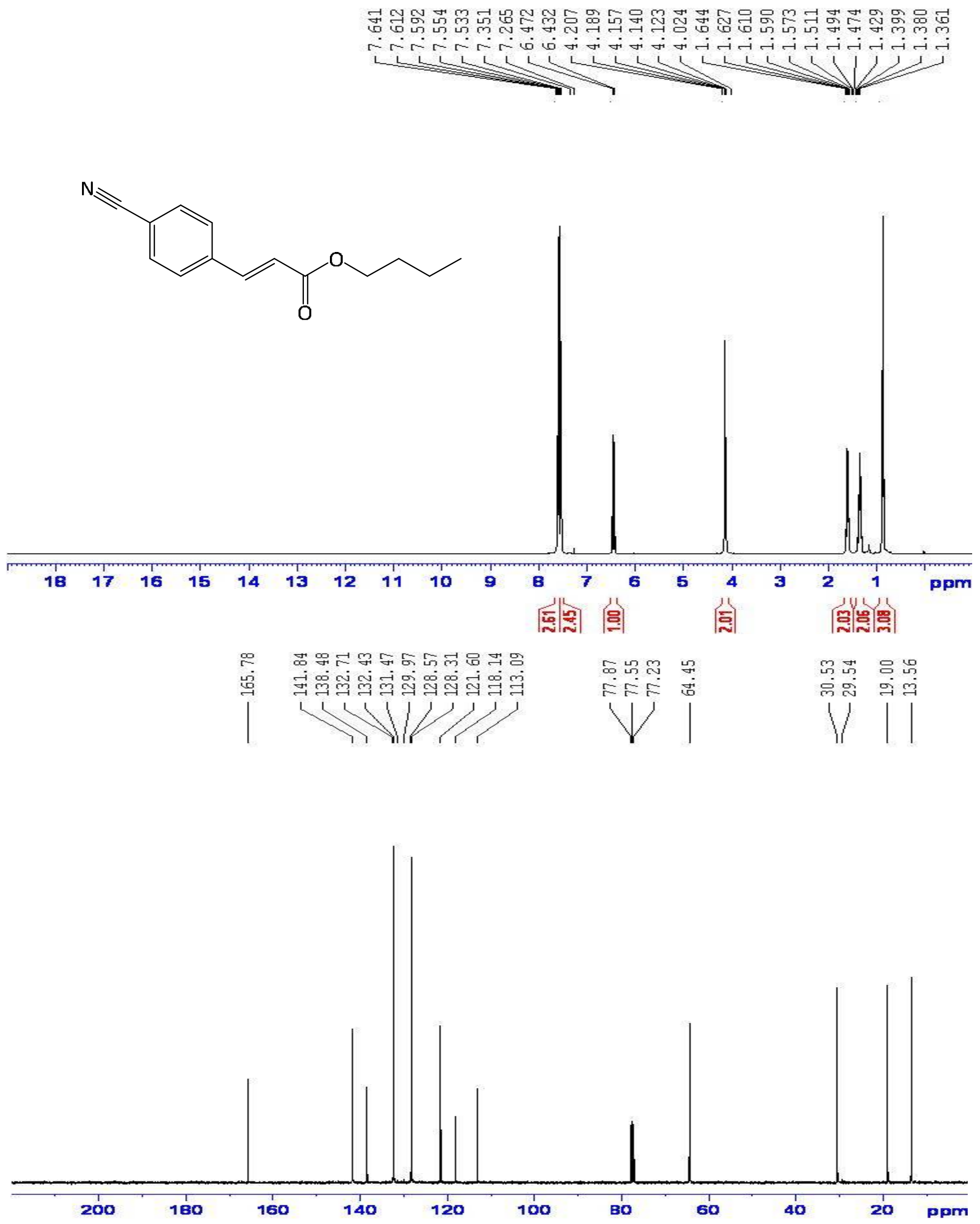


**Figure S8:** $^1$H NMR and $^{13}$C NMR spectra of (E)-*n*-butyl 3-(4-cyanophenyl) acrylate

$^1$H NMR (400 MHz, $CDCl_3$): δ 7.60 (d, 2H, $^3J$ = 8.0 Hz), 7.57 (d, 1H, $^3J$ = 15.2 Hz), 7.54 (d, 2H, $^3J$ = 8.4 Hz), 6.45 (d, 1H, $^3J$ = 16.0 Hz), 4.14 (t, 2H, $^3J$ = 6.8 Hz), 1.57-1.64 (m, 2H), 1.30-1.38 (m, 2H), 0.77 (t, 3H, $^3J$ = 7.0 Hz) ppm. $^{13}$C NMR (100 MHz, $CDCl_3$), δ 165.7, 141.8, 138.4, 132.4, 128.3, 121.6, 118.1, 113.0, 64.4, 30.5, 19.0, 13.5 ppm.

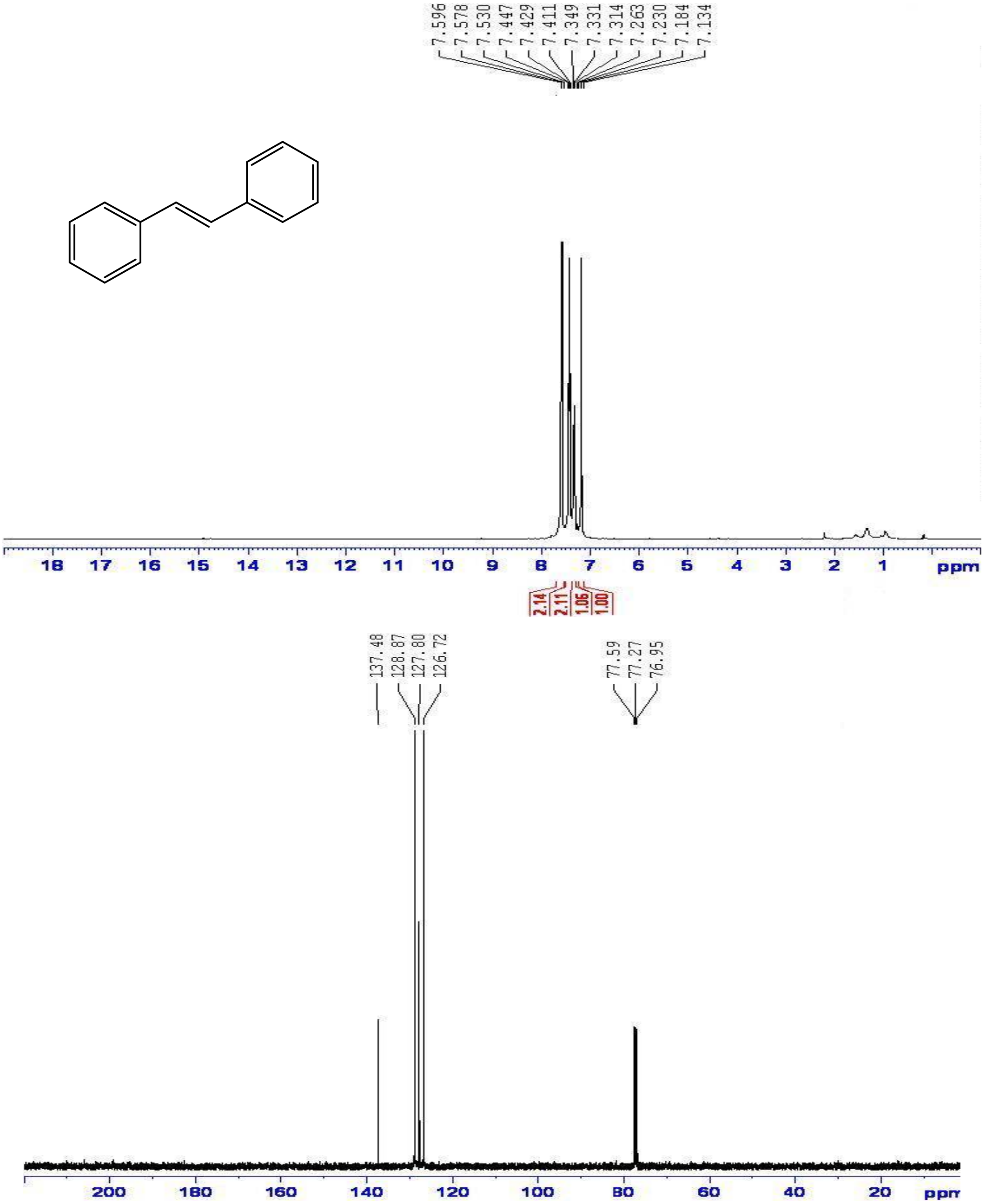


**Figure S9:** $^{1}H$ NMR and $^{13}C$ NMR spectra of (E)-1,2-diphenylethene

$^{1}H$ NMR (400 MHz, $CDCl_3$): $\delta$ 7.58 (d, 4H, $^{3}J$ = 7.2 Hz), 7.42 (t, 4H, $^{3}J$ = 7.0 Hz), 7.33 (t, 2H, $^{3}J$ = 6.8 Hz), 7.18 (s, 2H) ppm. $^{13}C$ NMR (100 MHz, $CDCl_3$), δ 137.4, 128.8, 127.8, 126.7 ppm.

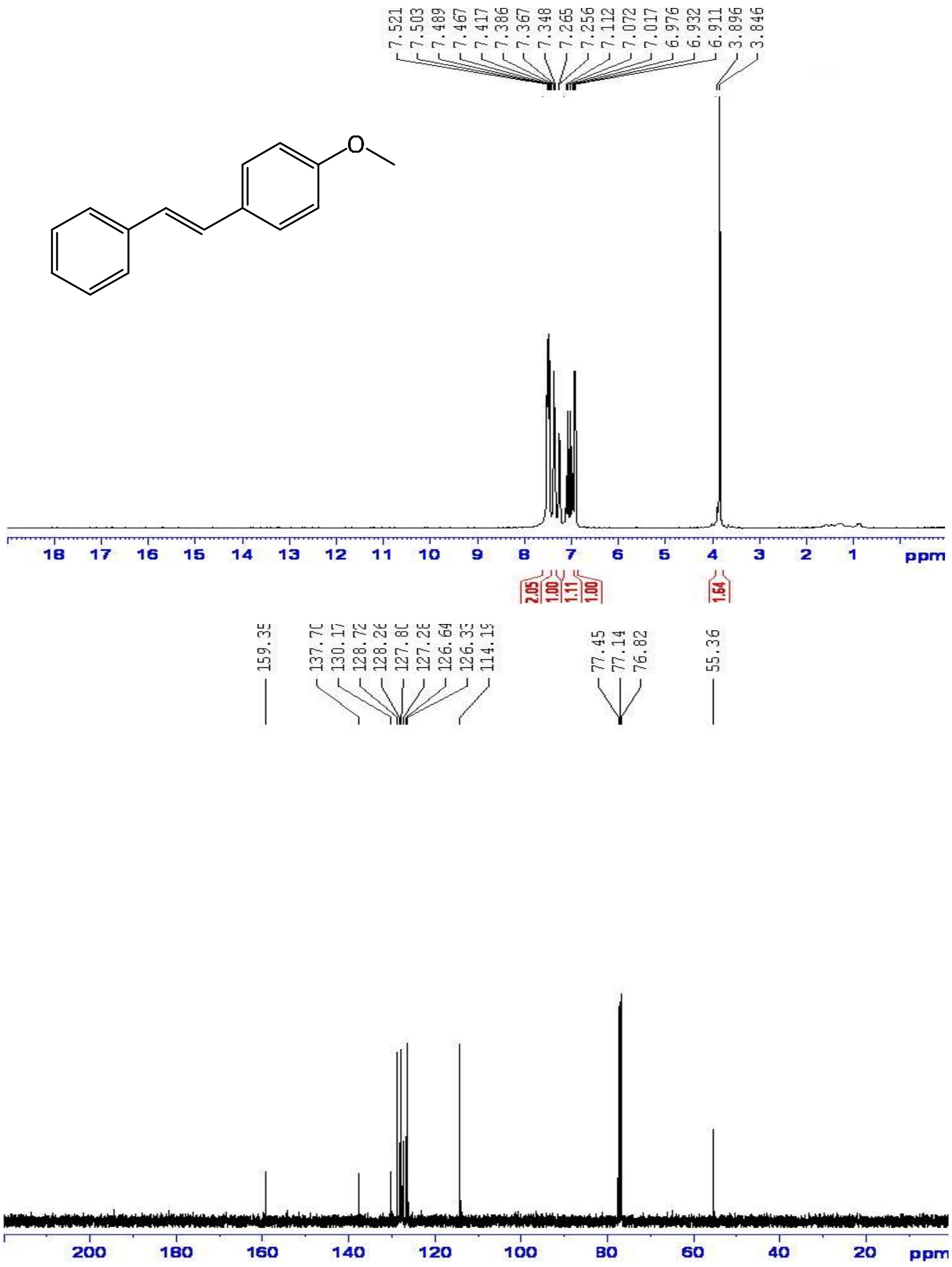


**Figure S10:** $^{1}$H NMR and $^{13}$C NMR spectra of (E)-1-methoxy-4-styrylbenzene

$^{1}$H NMR (400 MHz, $CDCl_3$): $\delta$ 7.46-7.52 (m, 3H), 7.36 (t, 2H, $^{3}J$ = 7.6 Hz), 7.09 (d, 2H, $^{3}J$ = 16.0 Hz), 6.99 (d, 2H, $^{3}J$ = 16.4 Hz), 6.92 (d, 2H, $^{3}J$ = 8.4 Hz), 3.89 (s, 3H) ppm. $^{13}$C NMR (100 MHz, $CDCl_3$), $\delta$ 159.3, 137.7, 130.1, 128.7, 128.2, 127.8, 127.2, 126.6, 126.3, 114.1, 55.3 ppm.

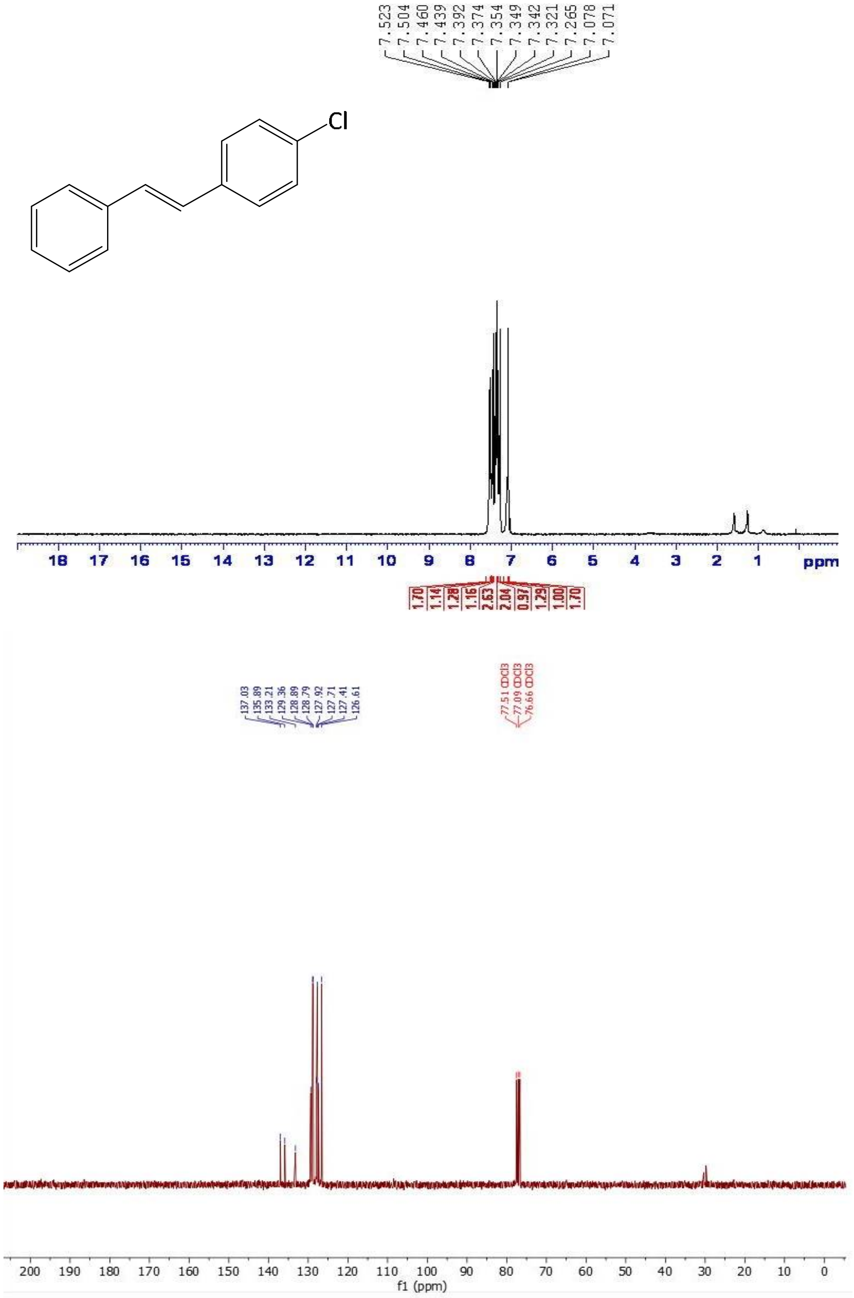


**Figure S11:** $^{1}H$ NMR and $^{13}C$ NMR spectra of (E)-1-chloro-4-styrylbenzene

$^{1}H$ NMR (400 MHz, $CDCl_3$): $\delta$ 7.50-7.52 (m, 2H), 7.43-7.46 (m, 2H), 7.28-7.39 (m, 5H), 7.07 (s, 2H) ppm.

$^{13}C$ NMR (75 MHz, $CDCl_3$), $\delta$ 137.0, 135.8, 133.2, 129.3, 128.8, 128.7, 127.9, 127.7, 127.4, 126.6 ppm.

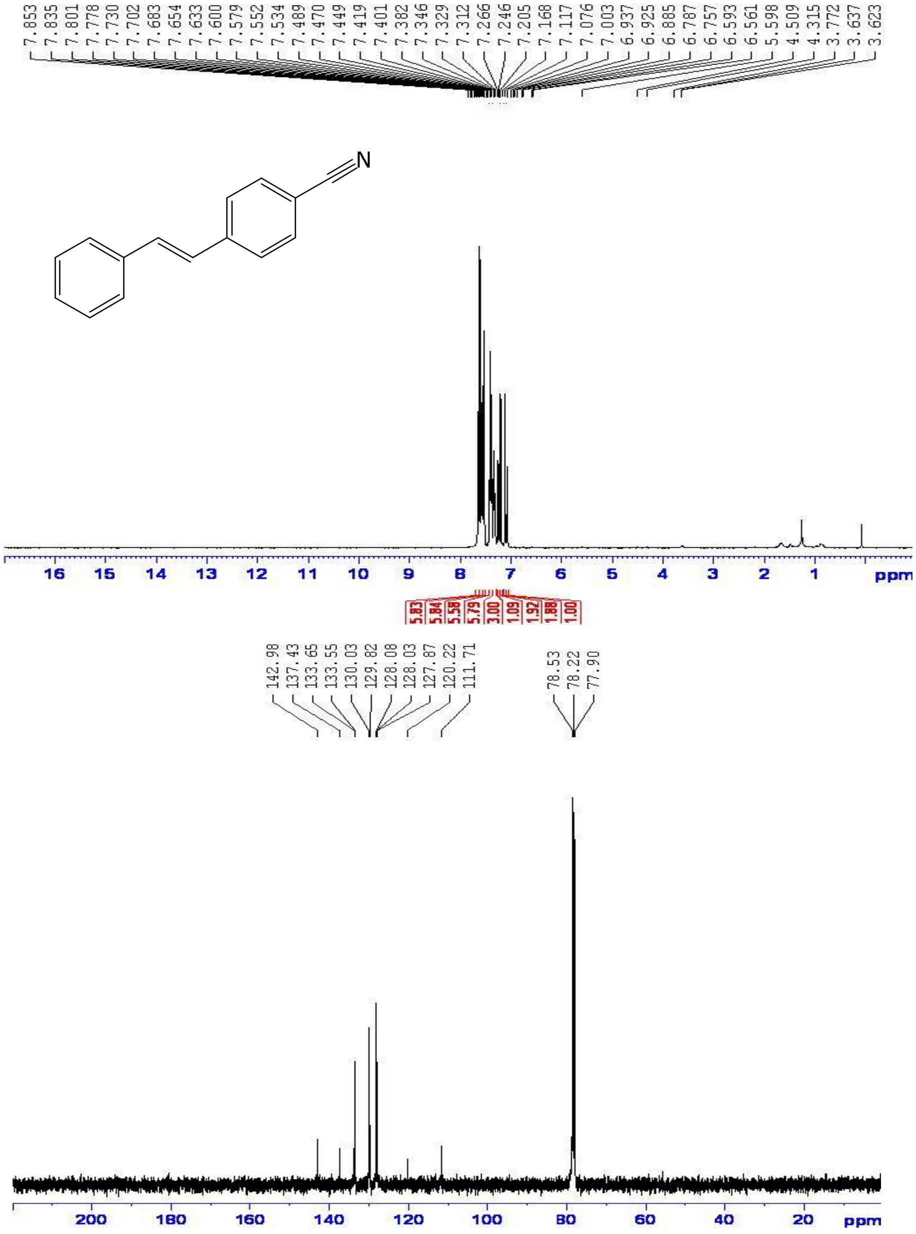


**Figure S12:** $^{1}H$ NMR and $^{13}C$ NMR spectra of (E)-4-styrylbenzonitrile

$^{1}H$ NMR (400 MHz, $CDCl_3$): δ 7.64 (d, 2H, $^{3}J$ = 8.4 Hz), 7.58 (d, 2H, $^{3}J$ = 8.4 Hz), 7.54 (d, 2H, $^{3}J$ = 7.2 Hz), 7.38-7.41 (t, 2H, $^{3}J$ = 4.0 Hz), 7.31-7.34 (t, 1H, $^{3}J$ = 4.0 Hz), 7.22 (d, 1H, $^{3}J$ = 16.0 Hz), 7.09 (d, 1H, $^{3}J$ = 16.0 Hz) ppm. $^{13}C$ NMR (100 MHz, $CDCl_3$), δ 142.9, 137.4, 133.6, 133.5, 130.0, 129.8, 128.1, 128.0, 127.8, 120.2, 111.7 ppm.

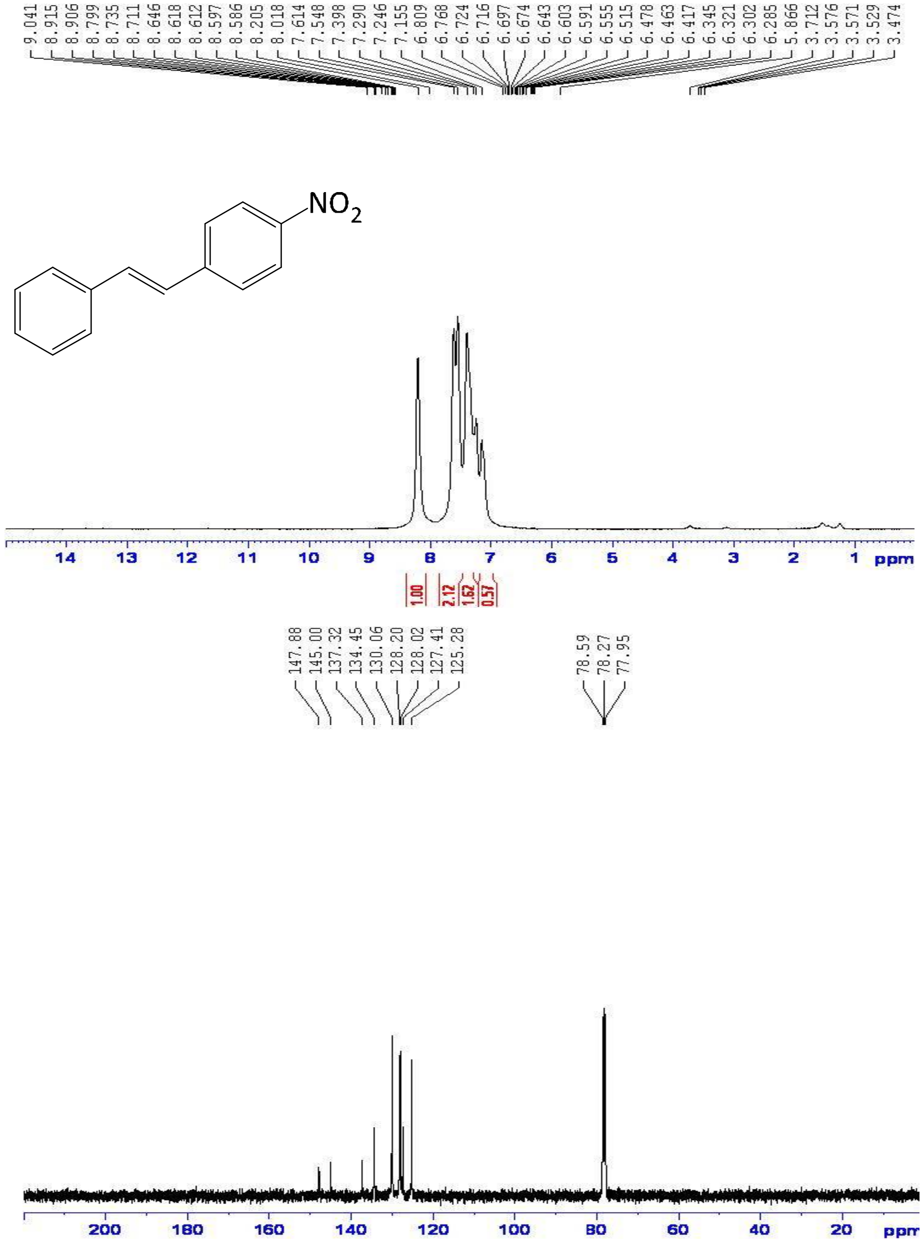


**Figure S13:** $^{1}H$ NMR and $^{13}C$ NMR spectra of (E)-1-nitro-4-styrylbenzene

$^{1}H$ NMR (400 MHz, $CDCl_3$): δ 8.15-8.26 (m, 2H), 7.49-766 (m, 4H), 7.40-7.45 (m, 4H), 7.06-7.18 (m, 1H) ppm. $^{13}C$ NMR (100 MHz, $CDCl_3$), δ 147.8, 145.0, 137.3, 134.4, 130.0, 128.2, 128.0, 127.4, 125.2 ppm.

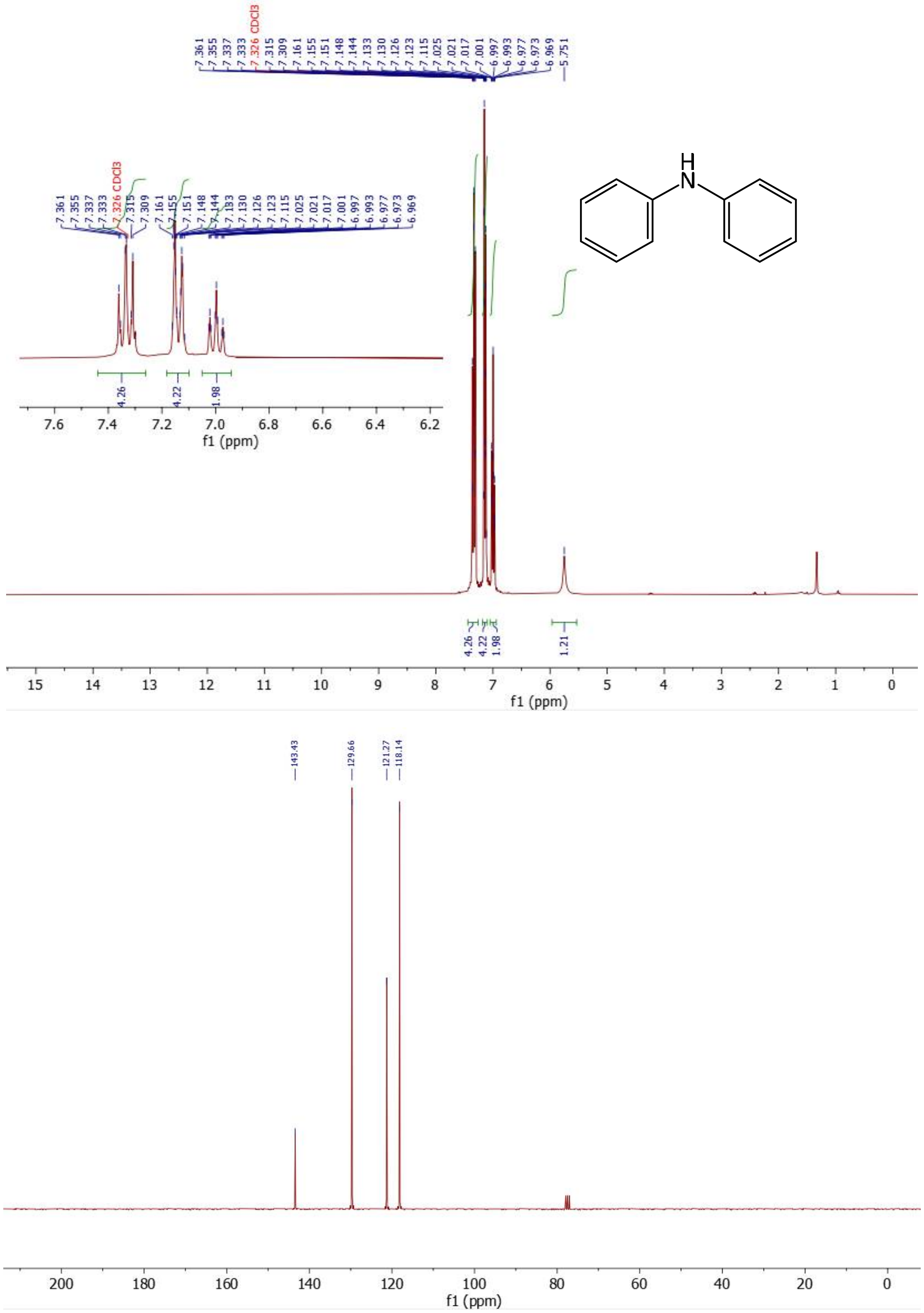


**Figure S14:** $^{1}H$ NMR and $^{13}C$ NMR spectra of diphenylamine

$^{1}H$ NMR (400 MHz, $CDCl_3$): δ 7.33 (t, 4H, *J* = 9.6 Hz), 7.15 (d, 4H, *J* = 10 Hz), 6.99 (d, 2H, *J* = 9.6 Hz), 5.75 (s, 1H) ppm. $^{13}C$ NMR (100 MHz, $CDCl_3$), δ 143.4, 129.6, 121.2, 118.1 ppm.

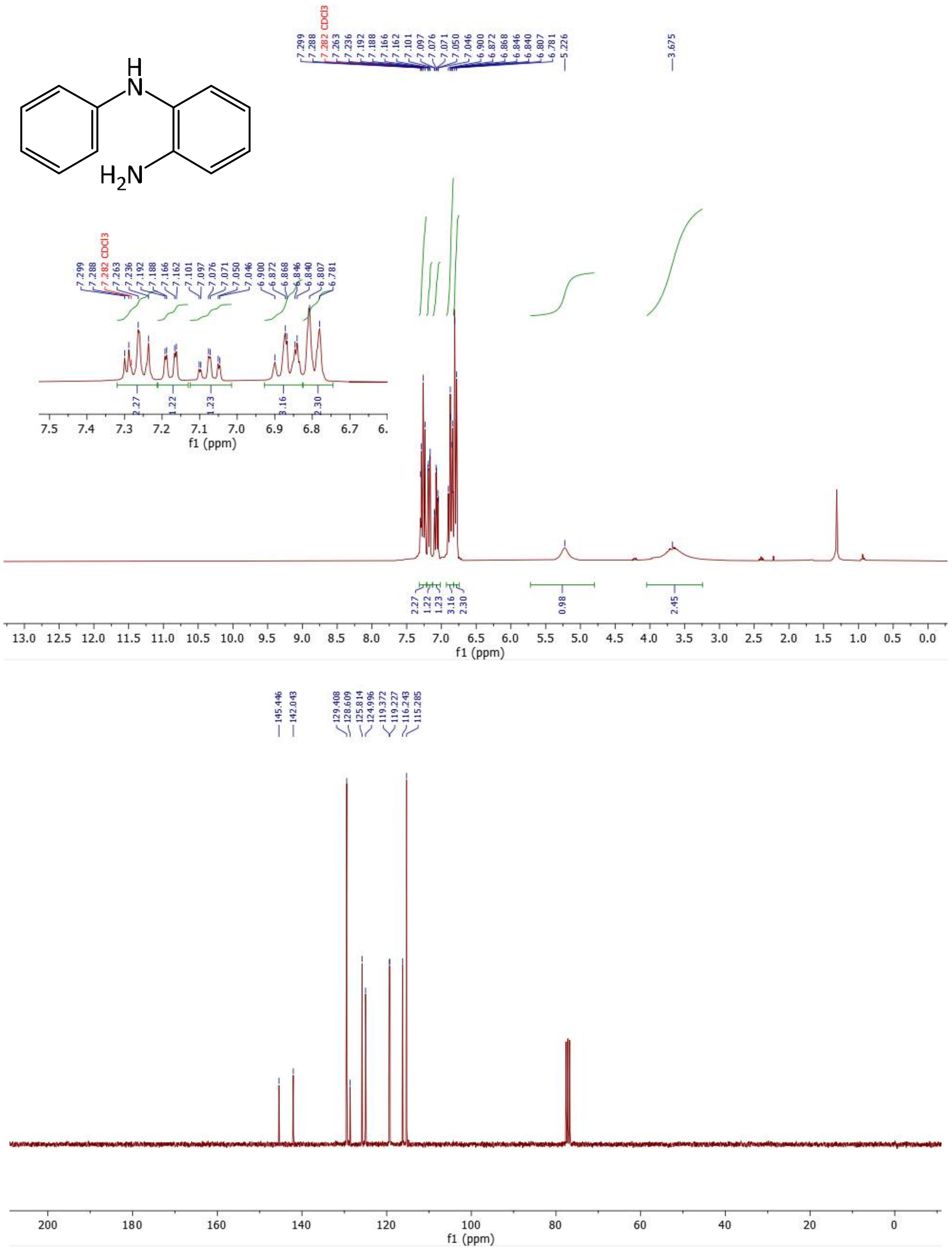


**Figure S15:** $^{1}$H NMR and $^{13}$C NMR spectra of 2-aminodiphenylamine

$^{1}$H NMR (400 MHz, $CDCl_3$): δ 7.29-7.23 (m, 2H), 7.18 (dd, 1H, *J* = 10.4 Hz), 7.07 (t, 1H, *J* = 10.4 Hz), 6.86 (t, 3H, *J* = 11.2 Hz), 6.80 (d, 2H, *J* = 10.4 Hz), 5.22 (s, 1H), 3.67 (s, 2H) ppm. $^{13}$C NMR (100 MHz, $CDCl_3$), δ 145.4, 142.0, 129.4, 128.6, 125.8, 124.9, 119.3, 119.2, 116.2, 115.2 ppm.

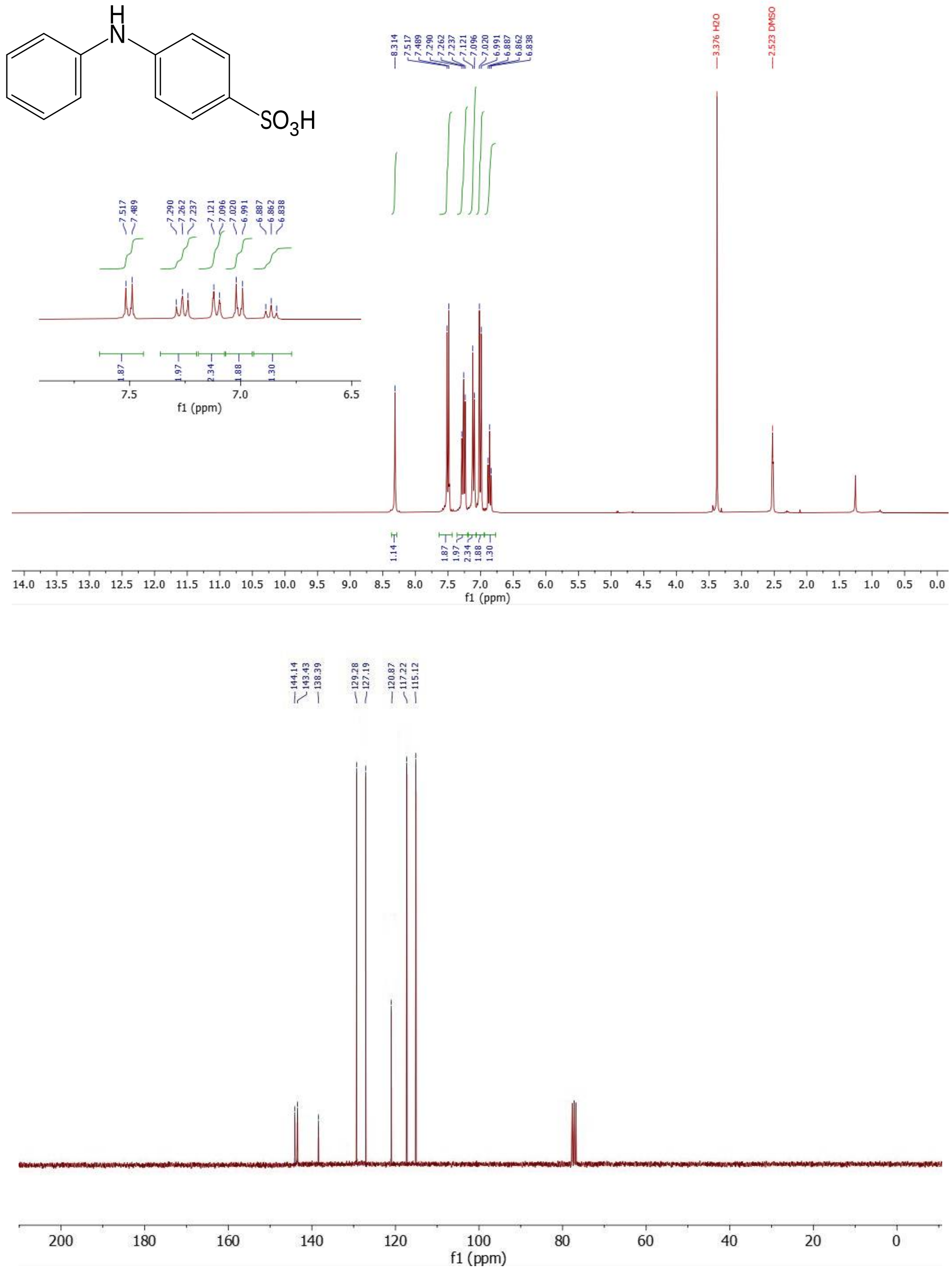


**Figure S16:** $^{1}H$ NMR and $^{13}C$ NMR spectra of 4-sulfodiphenylamine

$^{1}H$ NMR (400 MHz, DMSO): δ 8.3 (s, 1H), 7.51 (d, 2H, *J* = 11.2 Hz), 7.26 (t, 2H, *J* = 10.0 Hz), 7.12 (d, 2H, *J* = 10.0 Hz), 7.02 (d, 2H, *J* = 11.6 Hz), 6.86 (t, 1H, *J* = 9.6 Hz) ppm. $^{13}C$ NMR (100 MHz, DMSO), δ 144.1, 143.4, 138.3, 129.2, 127.1, 120.8, 117.2, 115.1 ppm.

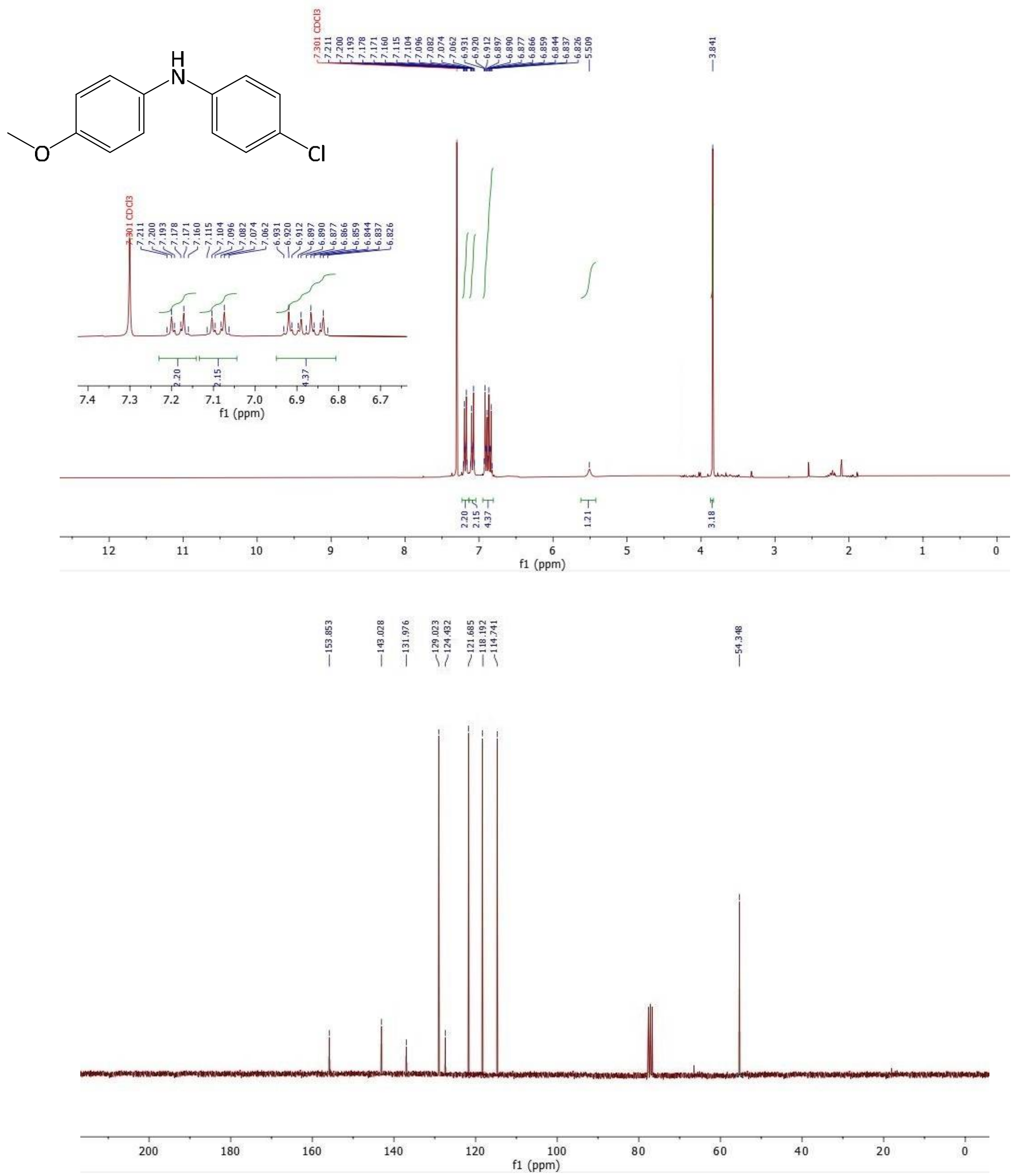


**Figure S17:** $^{1}$H NMR and $^{13}$C NMR spectra of 4-chloro-N-(4-methoxyphenyl)aniline

$^{1}$H NMR (400 MHz, $CDCl_3$): $\delta$ 7.2 (d, 2H, $J$ = 11.6 Hz), 7.1 (d, 2H, $J$ = 12 Hz), 6.9-6.8 (dd, 4H, $J$ = 12 Hz, $J$ = 11.6 Hz), 5.5 (s, 1H), 3.8 (s, 3H) ppm. $^{13}$C NMR (100 MHz, $CDCl_3$), $\delta$ 153.8, 143.0, 131.9, 129.0, 124.4, 121.6, 118.1, 114.7, 54.3 ppm.

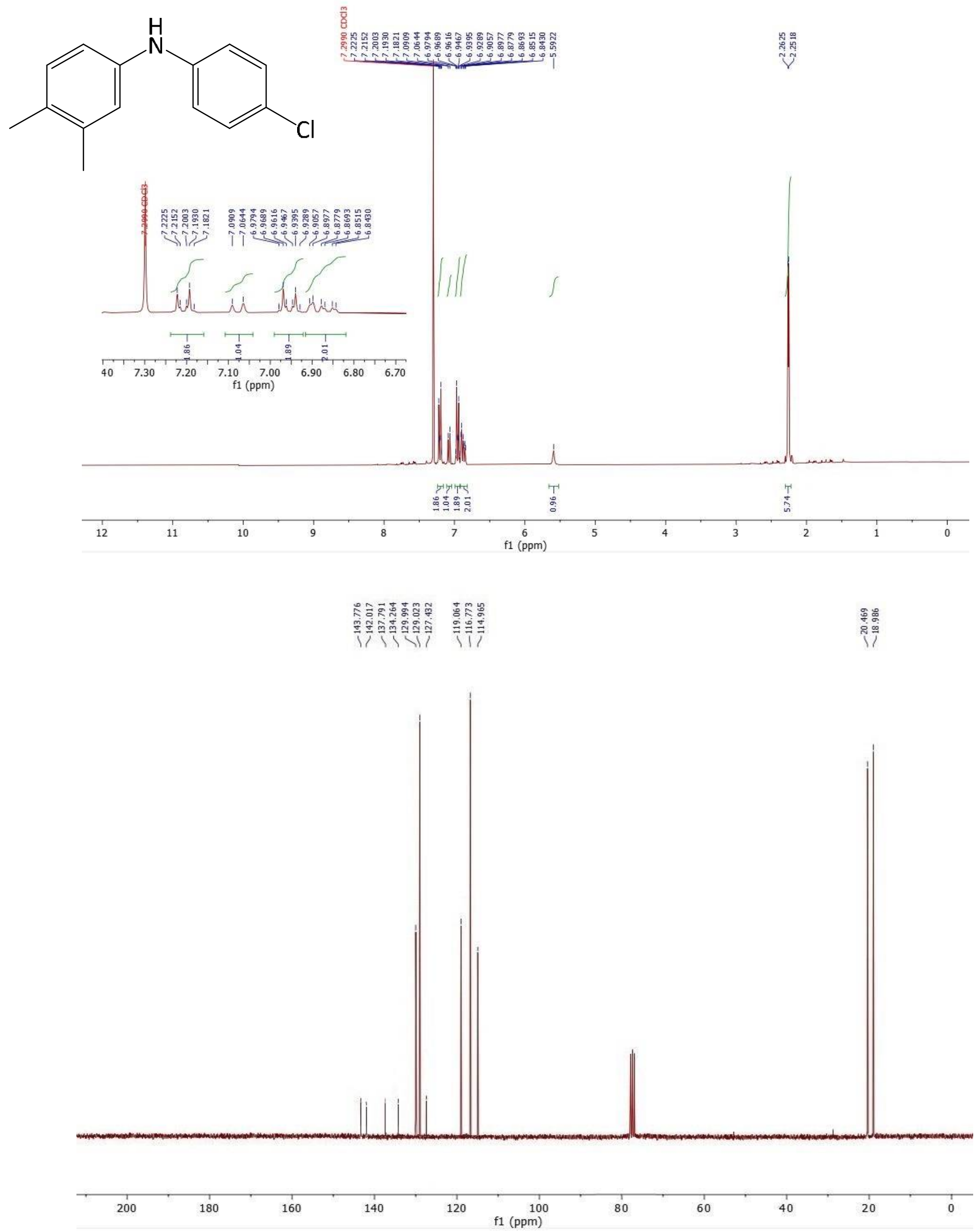


**Figure S18:** $^{1}$H NMR and $^{13}$C NMR spectra of N-(4-chlorophenyl)-3,4-dimethylaniline

$^{1}$H NMR (400 MHz, $CDCl_3$): δ 7.22 (d, 2H, *J* = 11.8 Hz), 7.09 (d, 1H, *J* = 10.6 Hz), 7.09 (d, 2H, *J* = 11.8 Hz), 6.87 (m, 2H), 5.59 (s, 1H) ppm. $^{13}$C NMR (100 MHz, $CDCl_3$), δ 143.7, 142.0, 137.7, 134.2, 129.9, 129.0, 127.4, 119.0, 116.7, 114.9, 20.4, 18.9 ppm.
